# Supplementary material for: A haplotype-resolved pangenome of the barley wild relative Hordeum bulbosum
Source: Nature. 2025 Jul 9;645(8080):429–38. doi: 10.1038/s41586-025-09270-x (PMC12422954; doi:10.1038/s41586-025-09270-x)
Supplement: Supplementary file 1 — Supplementary Figs. 1–38. [file 41586_2025_9270_MOESM1_ESM.pdf]

nature portfolio

https://doi.org/10.1038/s41586-025-09270-x

Supplementary information

A haplotype-resolved pangenome of the barley wild relative *Hordeum bulbosum*

In the format provided by the authors and unedited

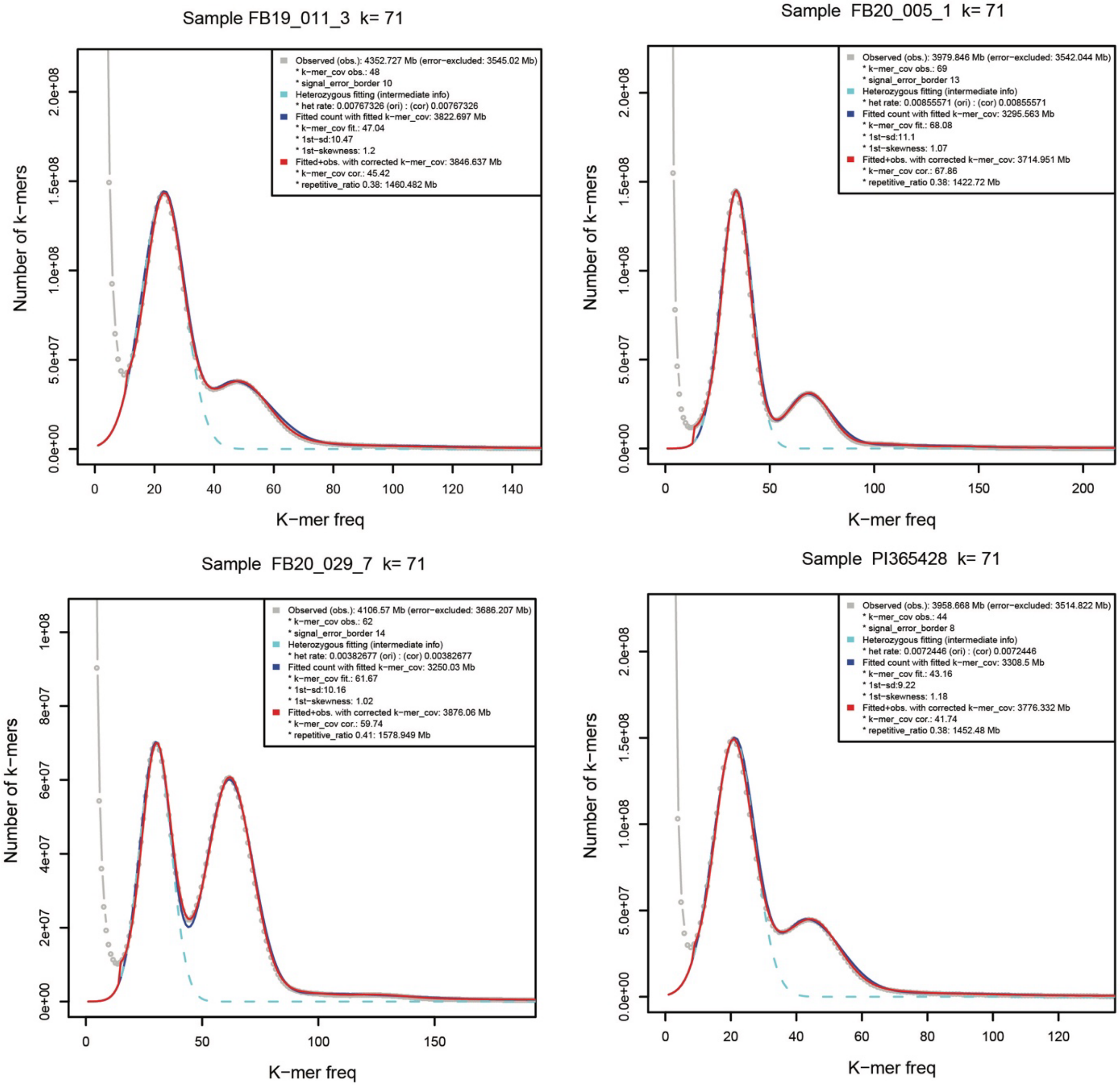

Supplementary Figure 1: K-mer analysis of 4 diploids with 71-mers.

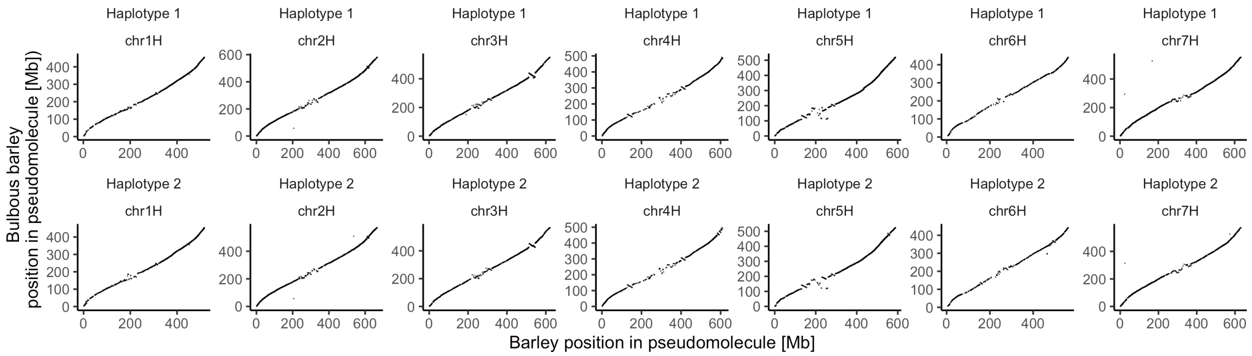

Supplementary Figure 2: Chromosome-level sequence alignments between haplotypes 1 (top) and 2 (bottom) of FB19-011-3 and the barley reference cultivar Morex.

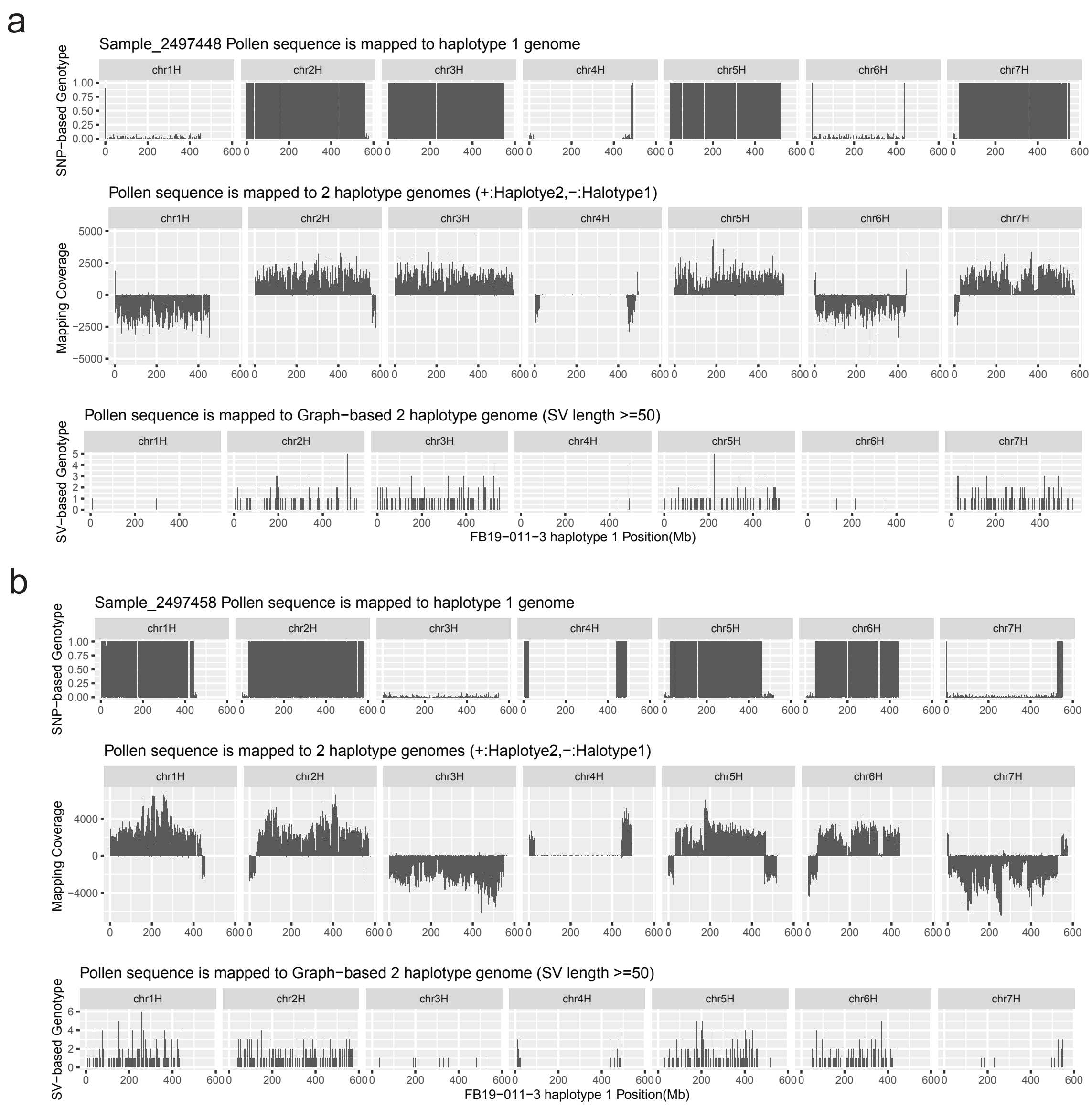

Supplementary Figure 3: Two examples of pollen sequencing genotyping, each illustrated with three methods. The upper panel shows genotyping results based on SNPs called against a haploid reference (FB19-011-3 haplotype 1), the middle panel depicts the read depth in alignments against a reference combing both parental haplotypes (FB19-011-3 haplotype 1 and 2); and the lower panel presents the graph-based approach using a pangenome graph constructed from the two parental haplotypes incorporating structural variants larger than 50bp. Data for two pollen nuclei are shown: **(a)** sample 2497448. **(b)** sample 2497458.

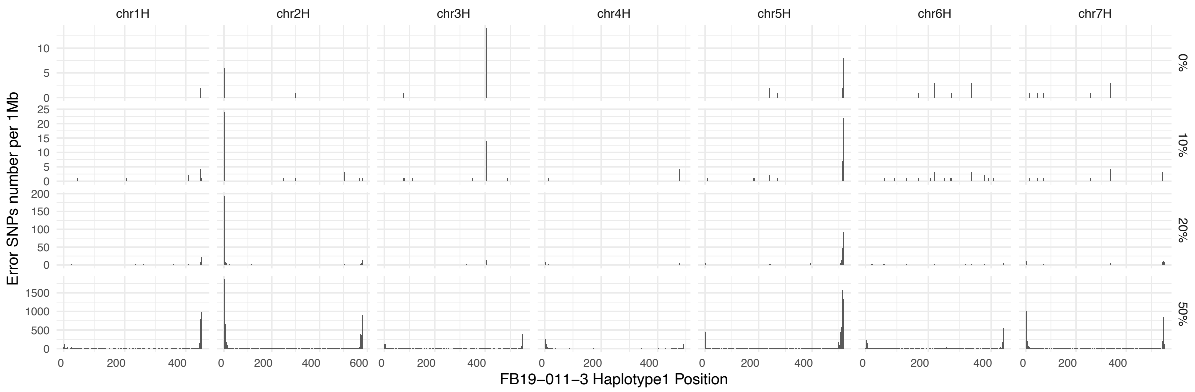

Supplementary Figure 4: Distribution of error SNPs at different recombination rates.

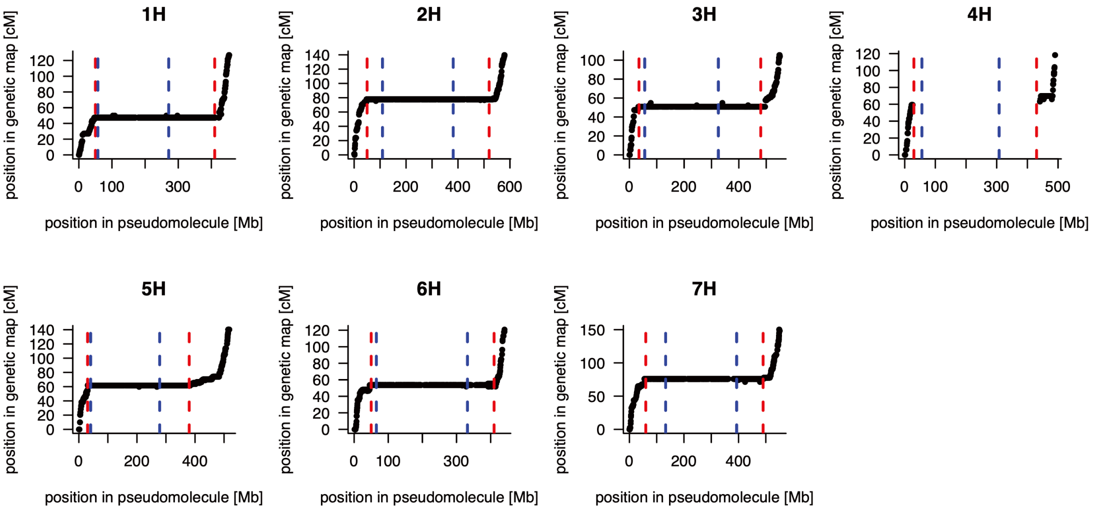

Supplementary Figure 5: Alignment between the chromosomal pseudomolecules of FB19-011-3 and the genetic map constructed from the pollen sequencing data. Dashed red lines indicate the boundaries of the non-recombining regions. The positions of *H. bulbosum* genes whose barley orthologs are close to the borders of the non-recombining regions in the latter species are marked by blue lines.

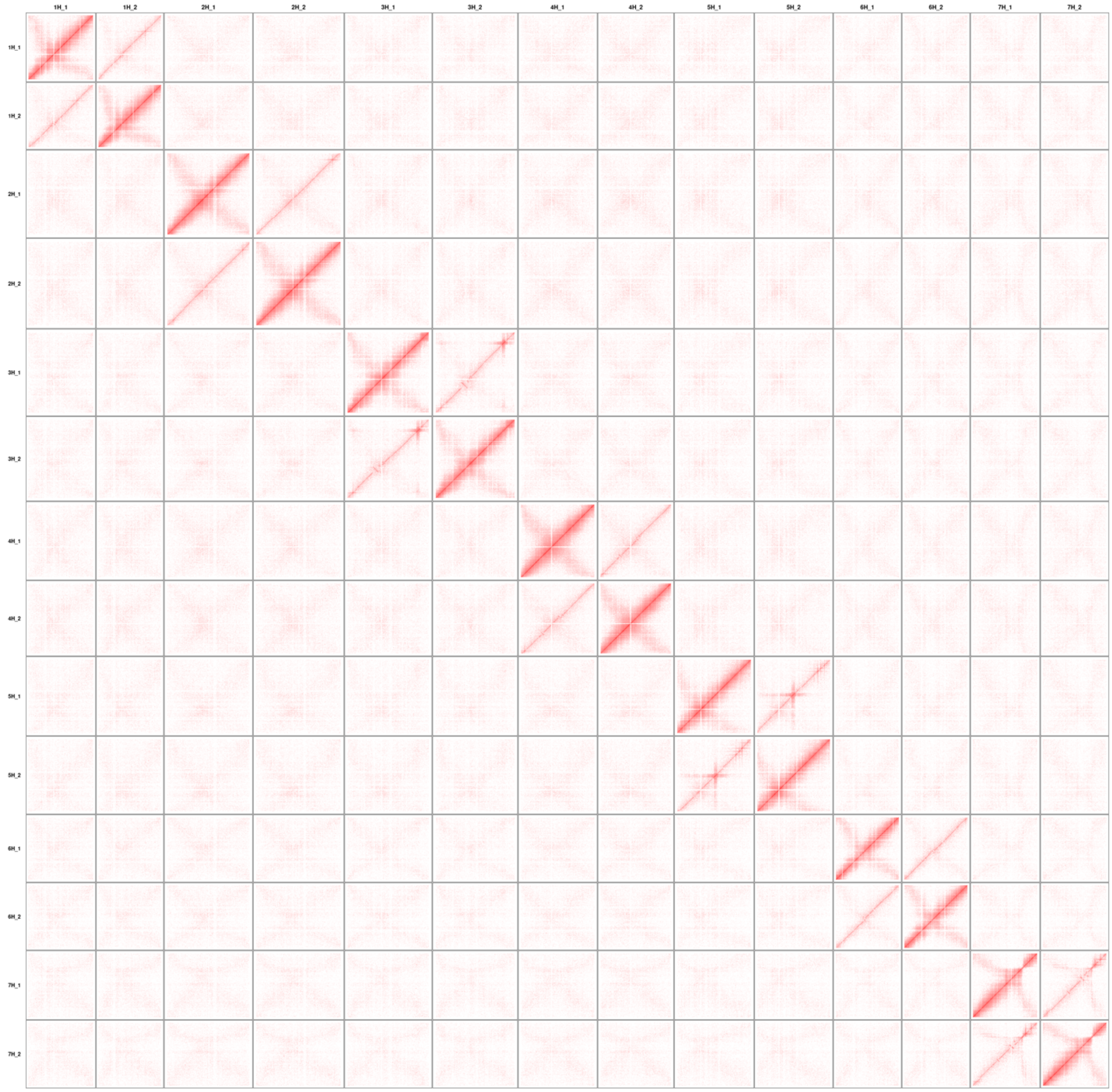

Supplementary Figure 6: FB20-005-1 Hi-C contact matrix. The intensity of pixels represents the normalized count of Hi-C links between 1 Mb windows on all chromosomes and haplotypes.

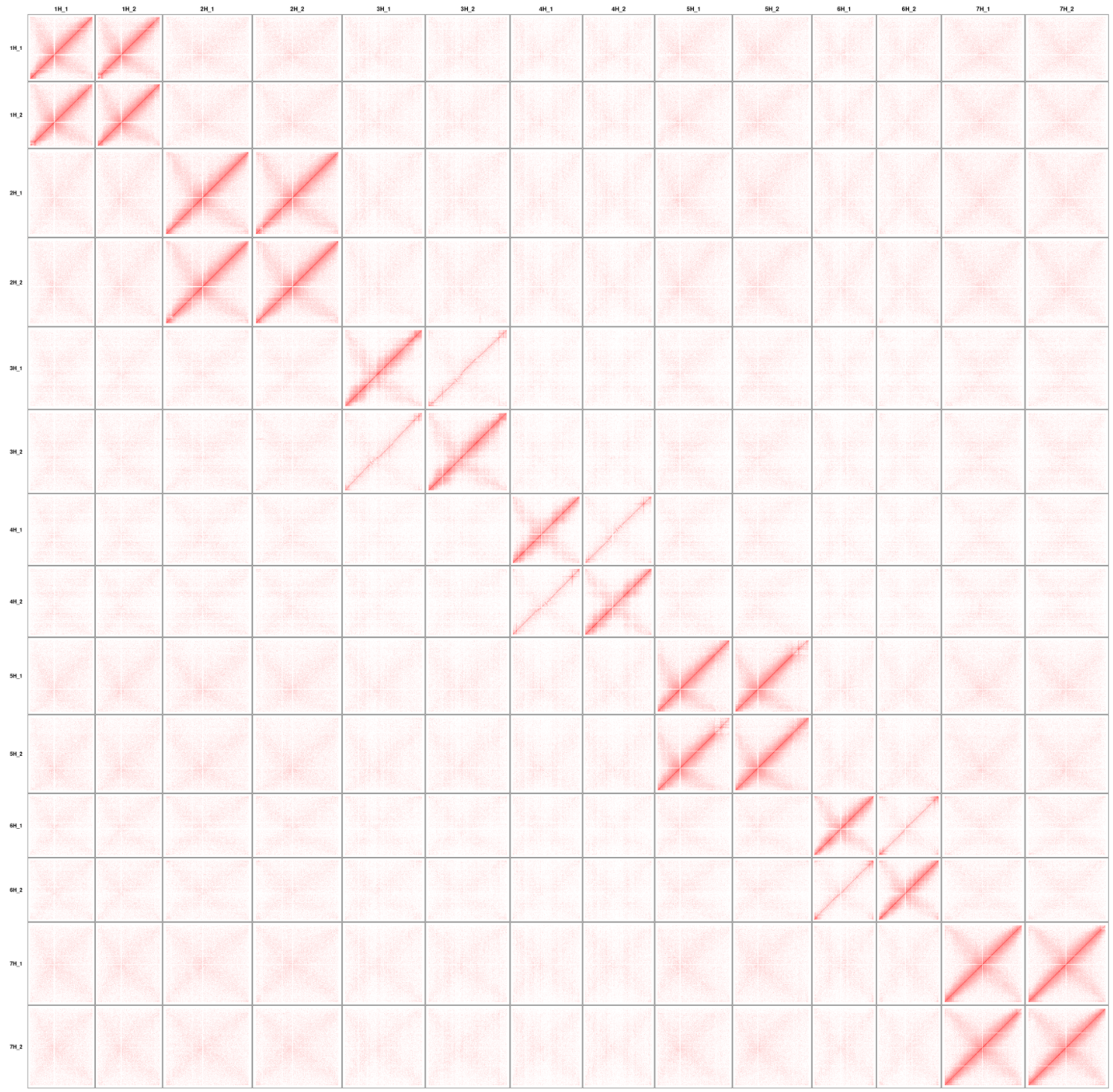

Supplementary Figure 7: FB20-029-7 Hi-C contact matrix.

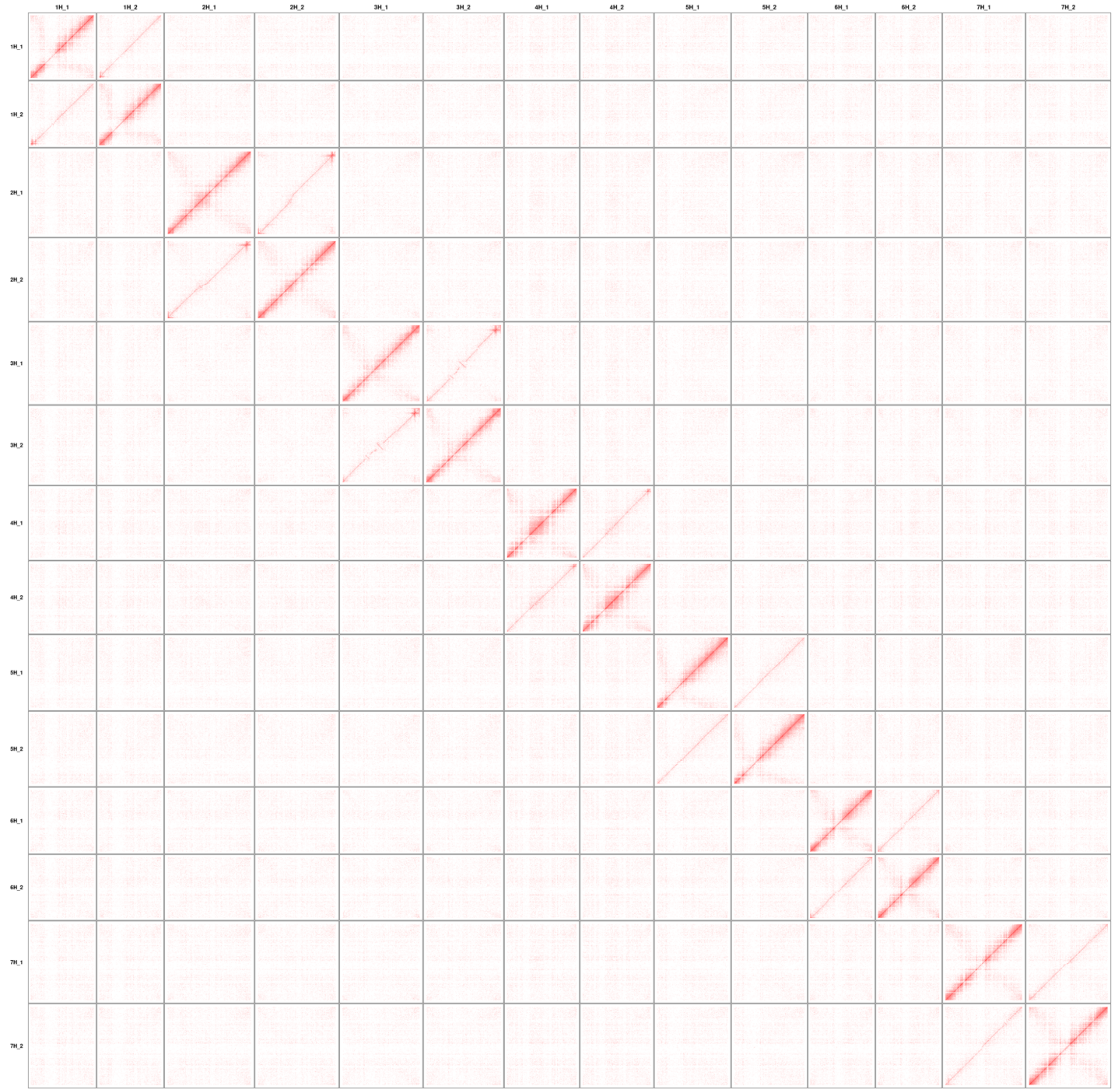

Supplementary Figure 8: PI365428 Hi-C contact matrix.

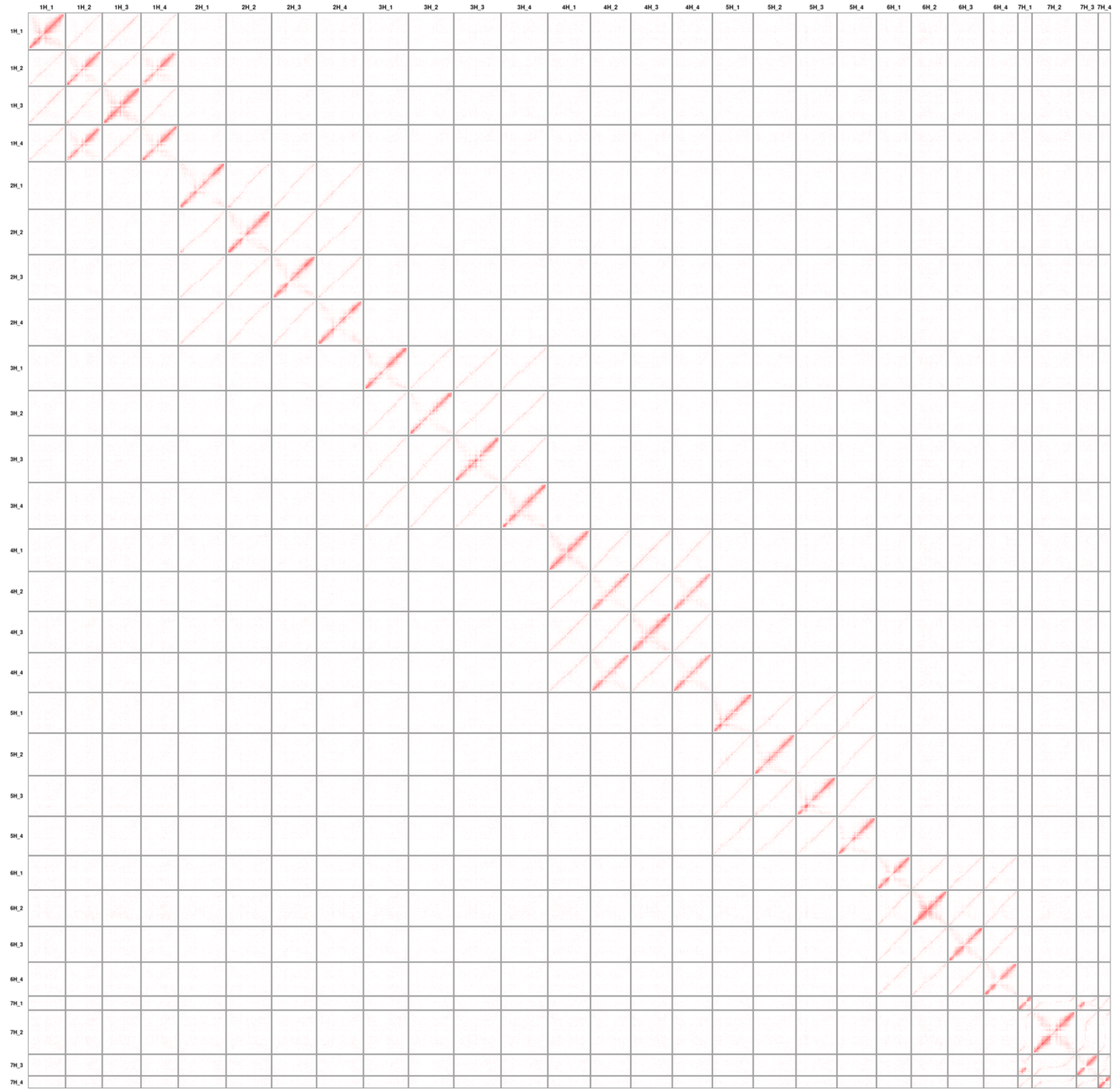

Supplementary Figure 9: FB19-001-1 Hi-C contact matrix.

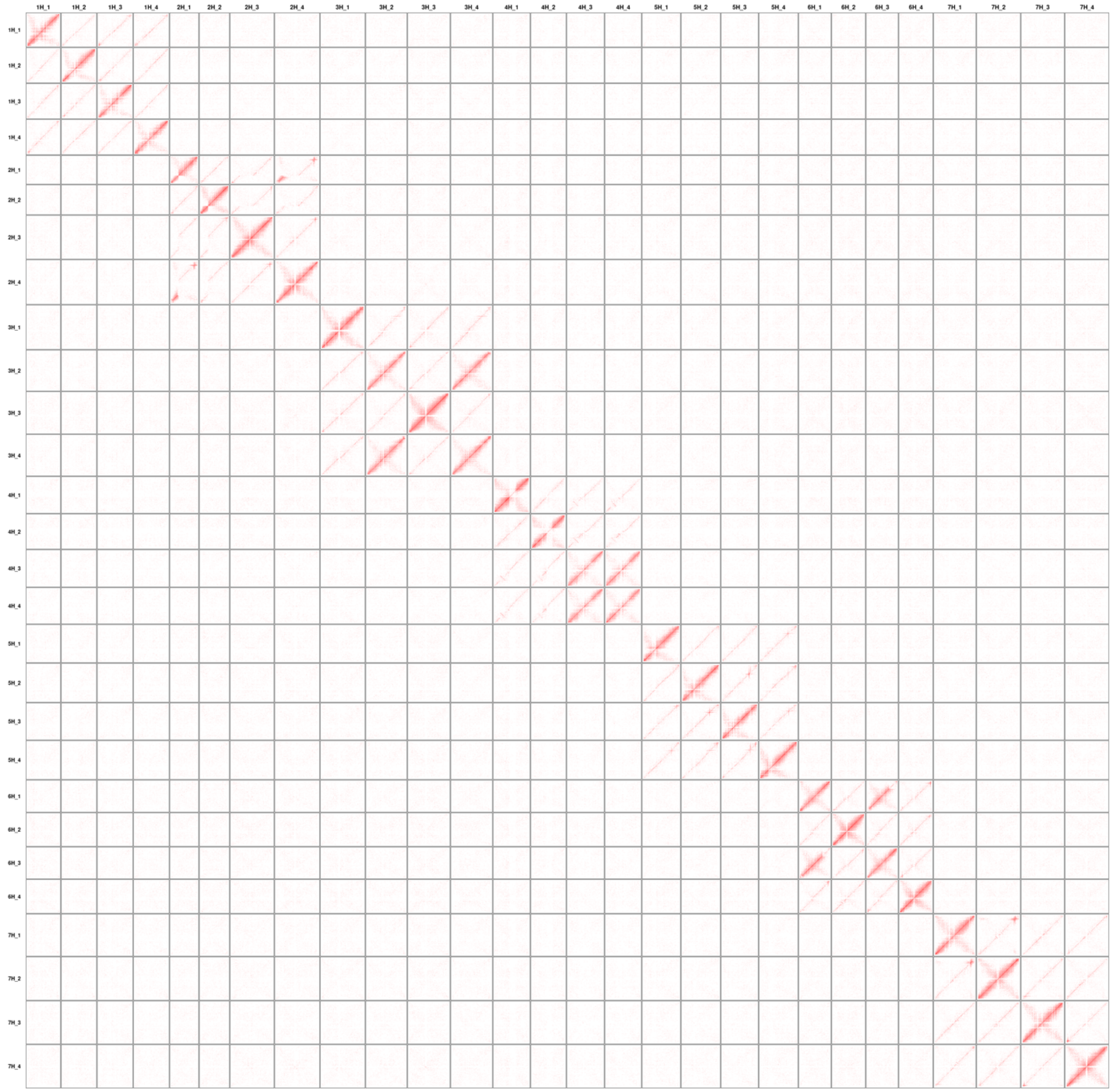

Supplementary Figure 10: GRA2256-1 Hi-C contact matrix.

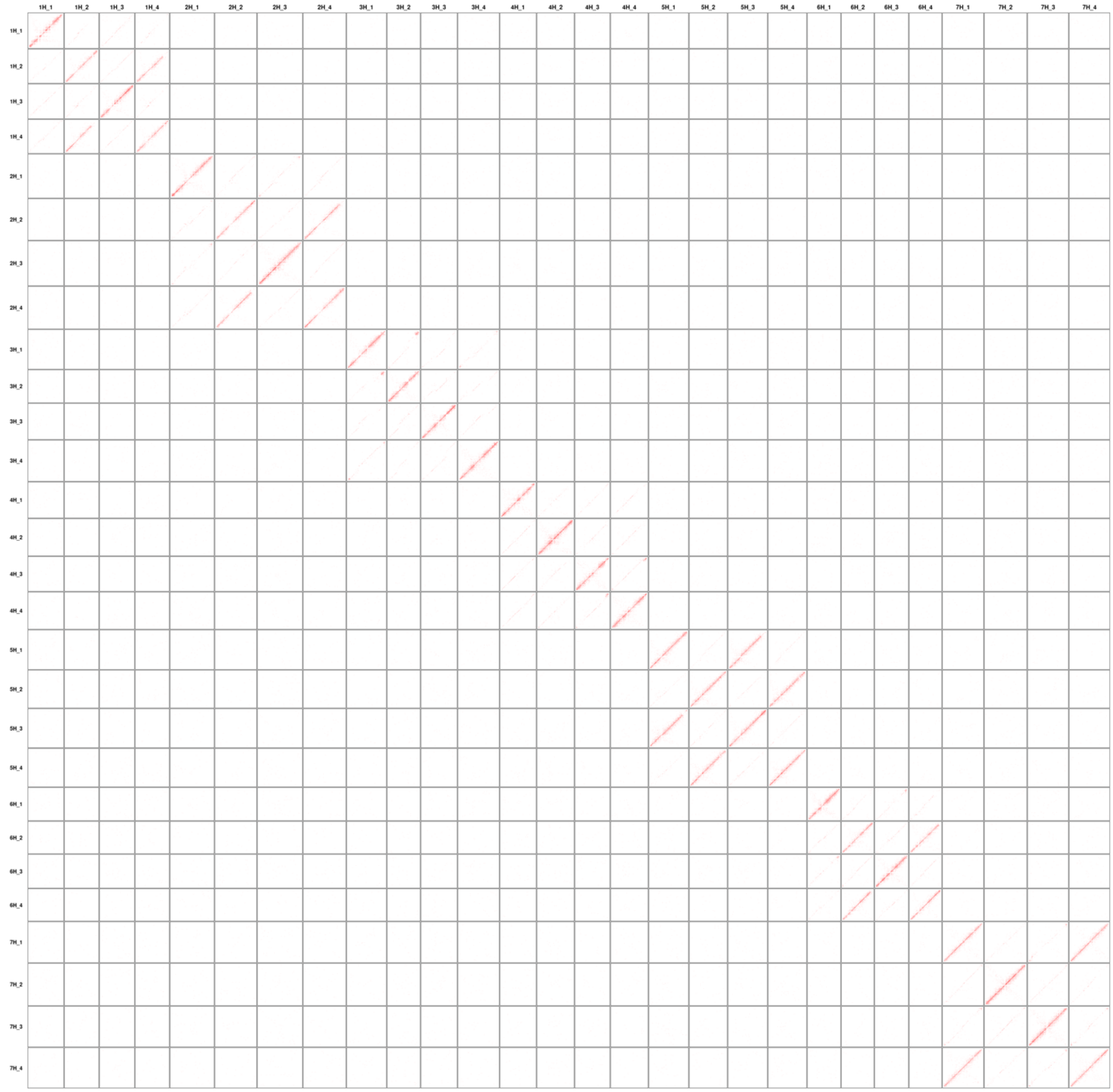

Supplementary Figure 11: A17 Hi-C contact matrix.

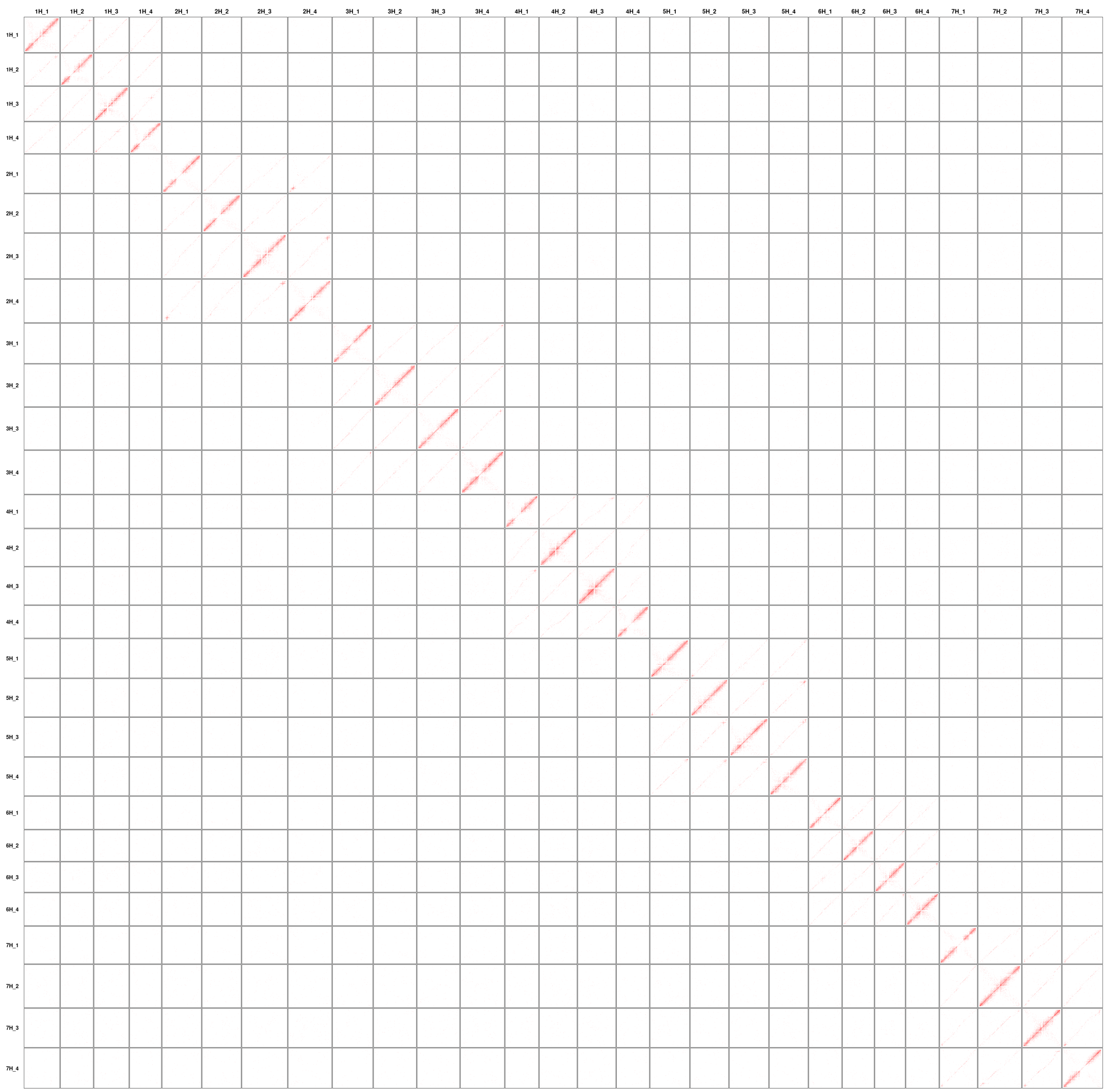

Supplementary Figure 12: A40 Hi-C contact matrix.

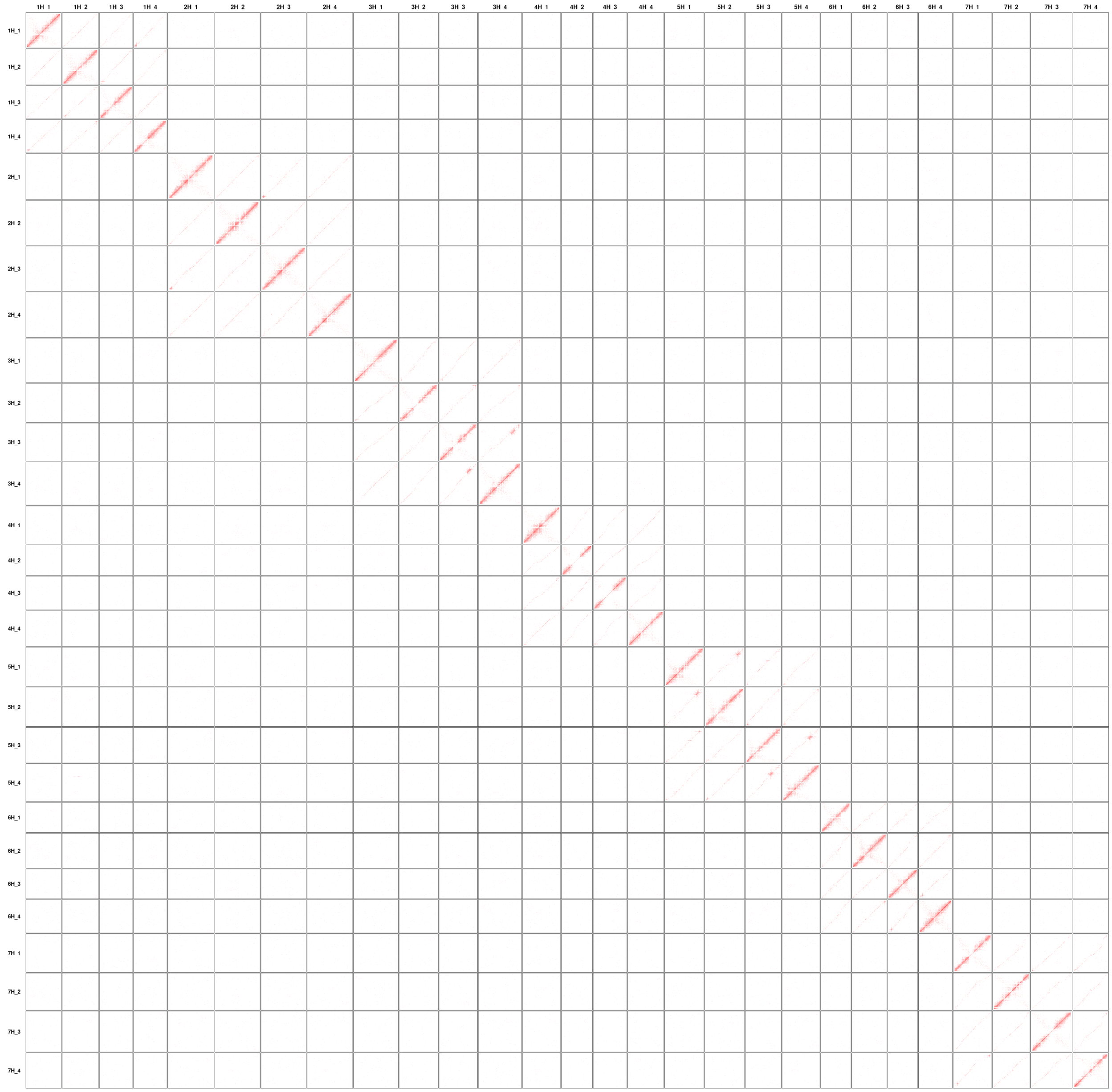

Supplementary Figure 13: A42 Hi-C contact matrix.

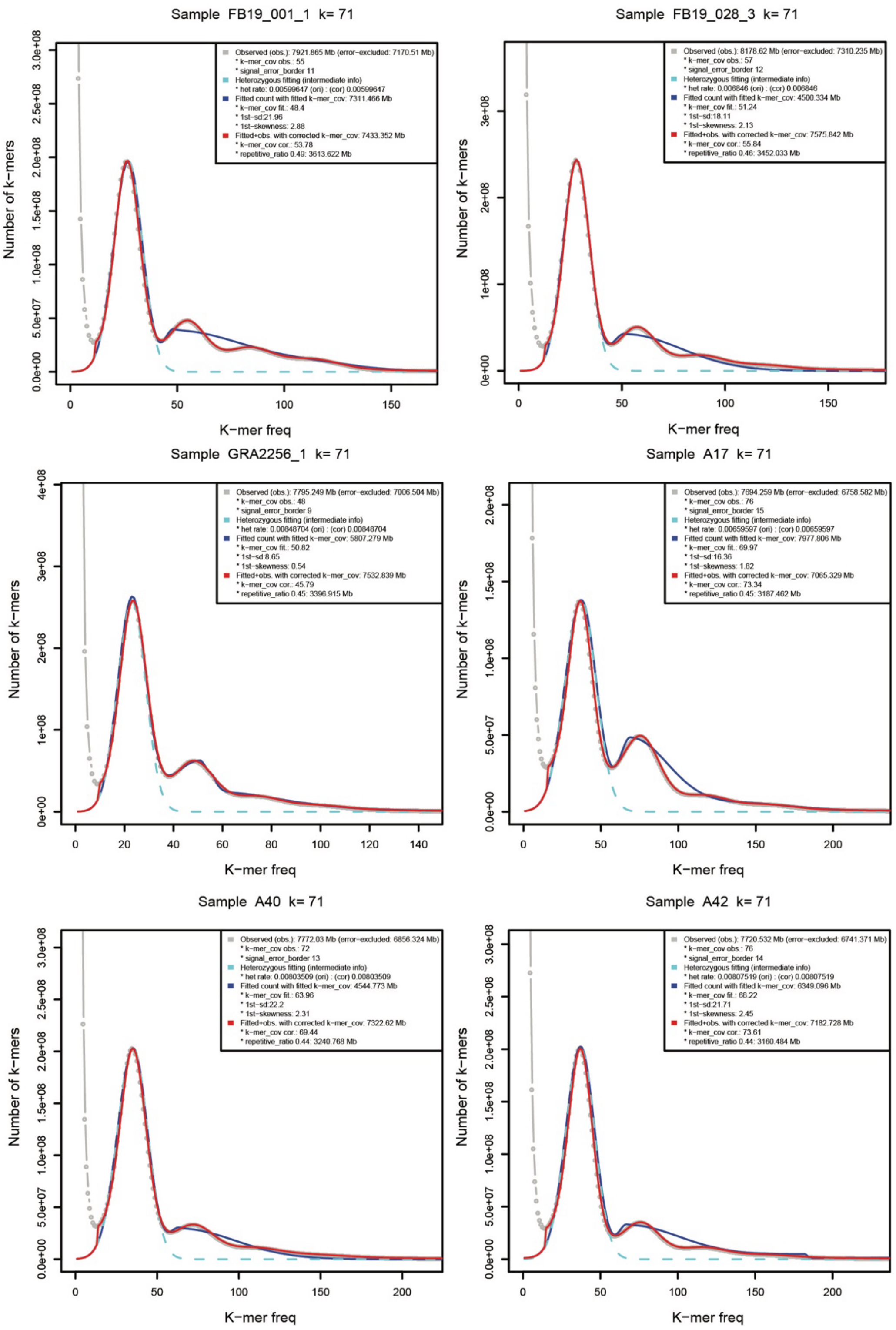

Supplementary Figure 14: K-mer analysis of 6 tetraploid genomes with 71-mers.

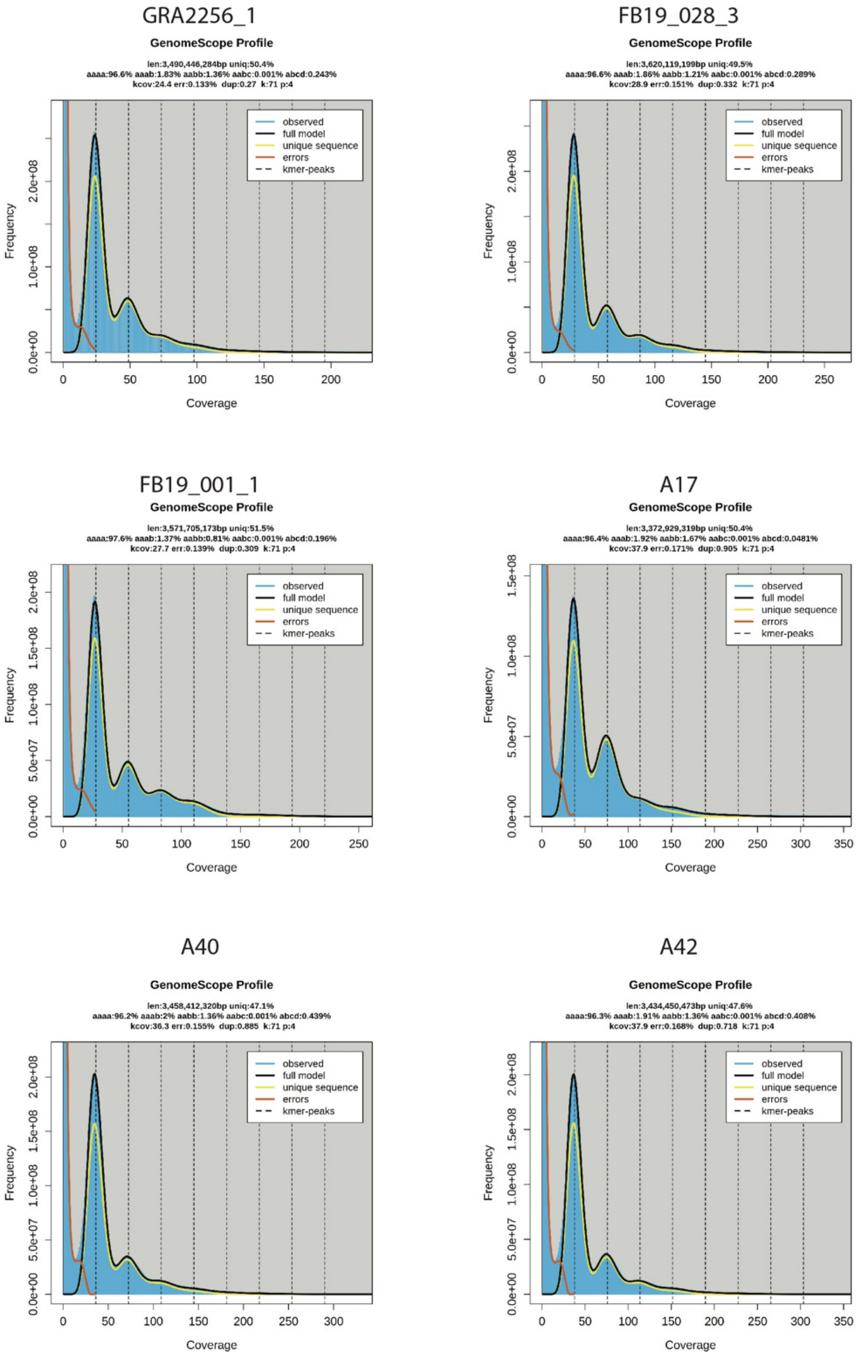

Supplementary Figure 15: K-mer frequency histograms of 6 tetraploid genomes with 71-mers computed with GenomeScope. Note that aaab > aabb, indicating autopolyploidy.

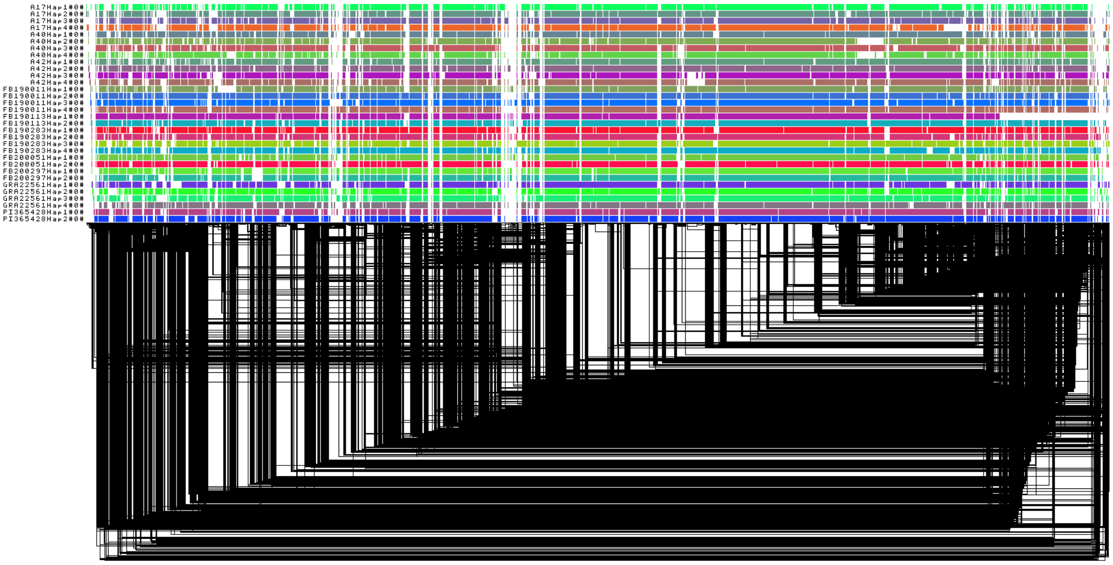

Supplementary Figure 16: ***H. bulbosum*** **chr1H graph pangenome constructed by the Minigraph-Cactus pangenome pipeline.**

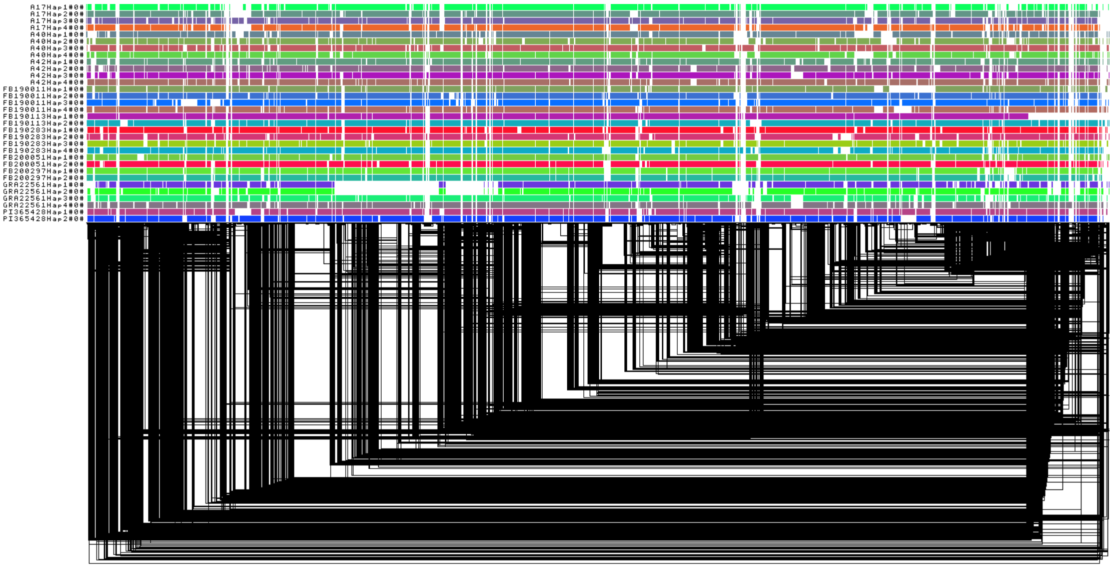

Supplementary Figure 17: ***H. bulbosum*** **chr2H graph pangenome constructed by the Minigraph-Cactus pangenome pipeline.**

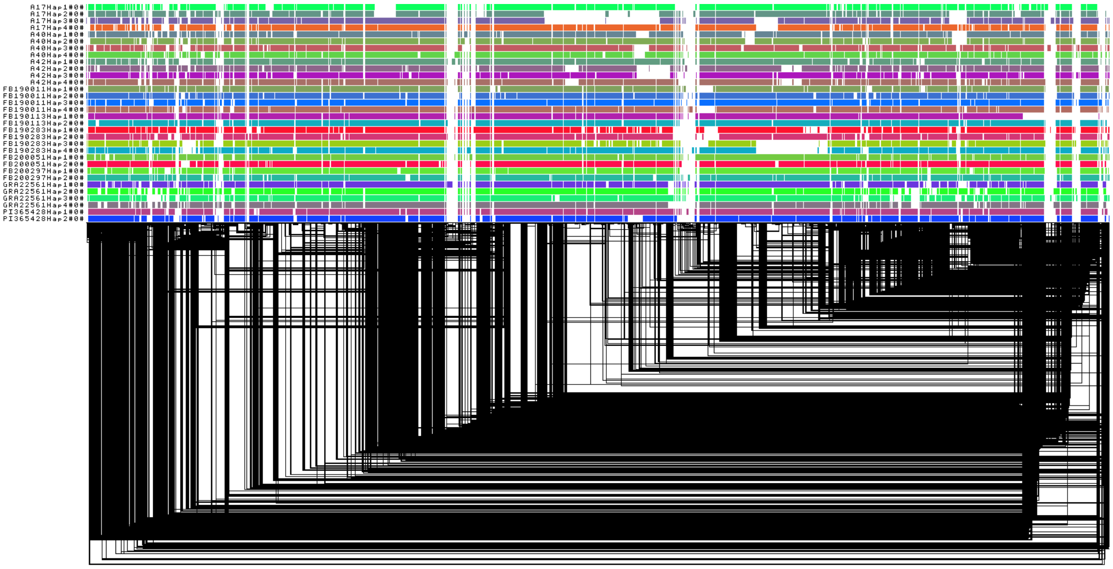

Supplementary Figure 18: ***H. bulbosum*** **chr3H graph pangenome constructed by the Minigraph-Cactus pangenome pipeline.**

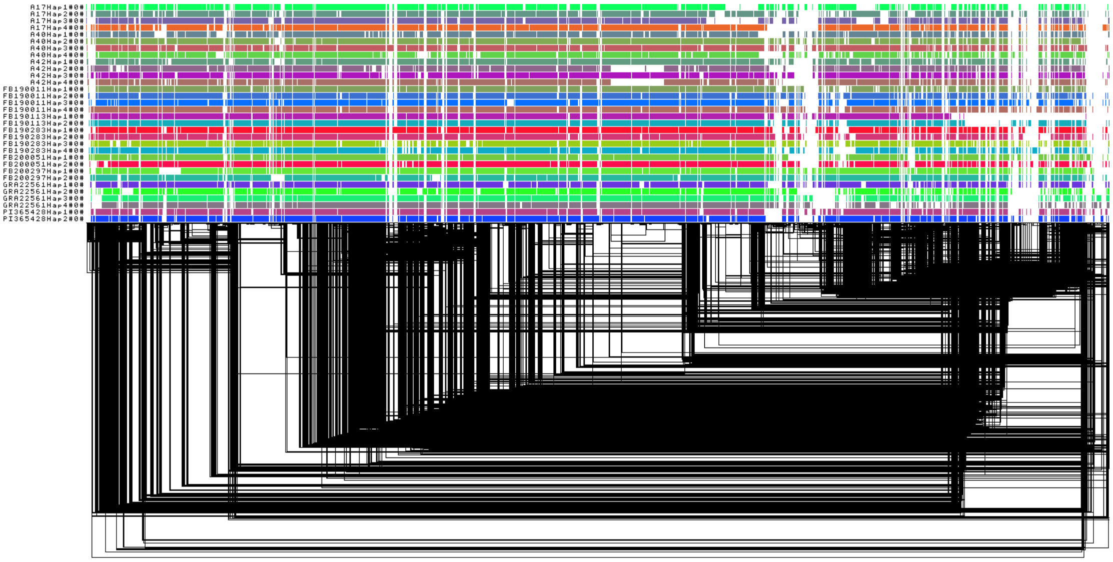

Supplementary Figure 19: ***H. bulbosum*** **chr4H graph pangenome constructed by the Minigraph-Cactus pangenome pipeline.**

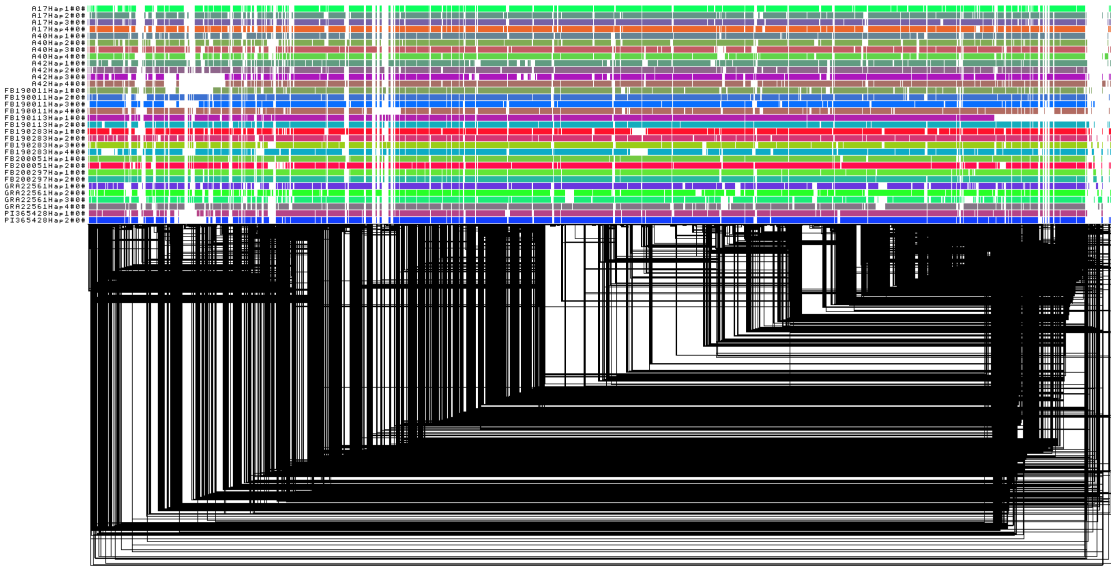

Supplementary Figure 20: ***H. bulbosum*** **chr5H graph pangenome constructed by the Minigraph-Cactus pangenome pipeline.**

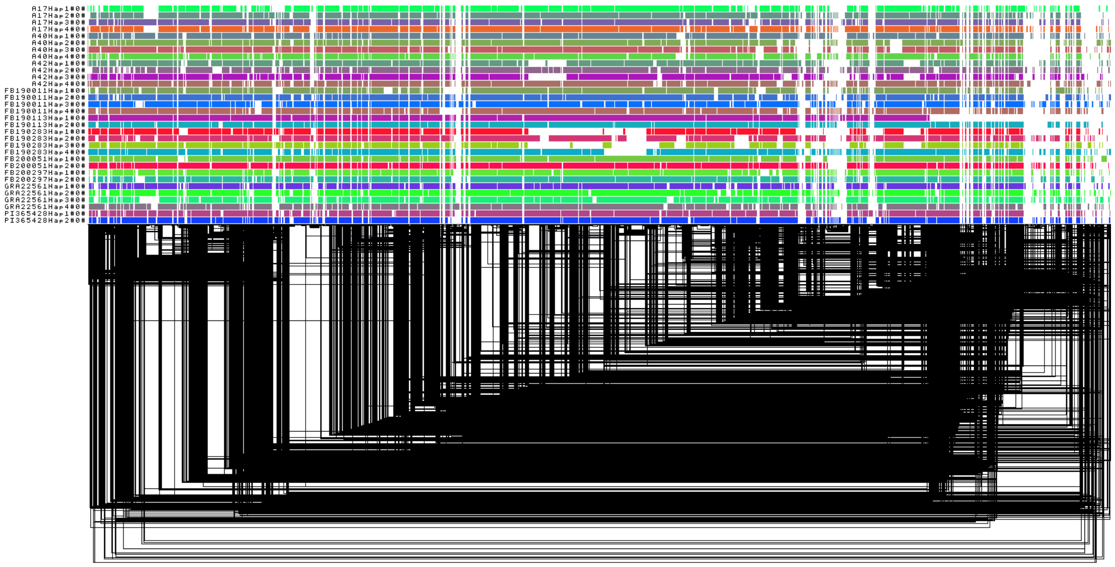

Supplementary Figure 21: ***H. bulbosum*** **chr6H graph pangenome constructed by the Minigraph-Cactus pangenome pipeline.**

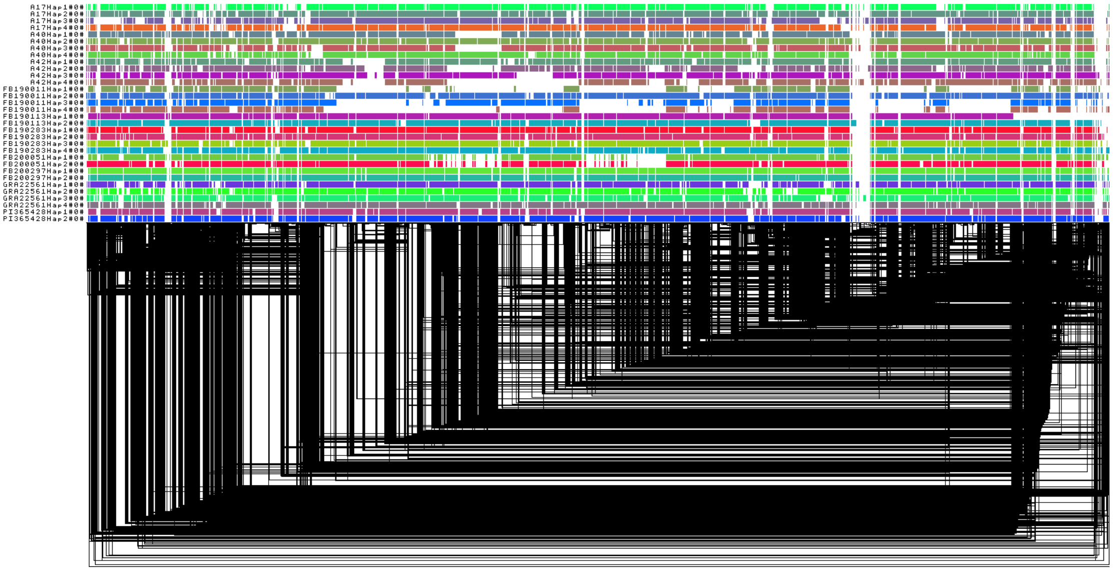

Supplementary Figure 22: ***H. bulbosum*** **chr7H graph pangenome constructed by the Minigraph-Cactus pangenome pipeline.**

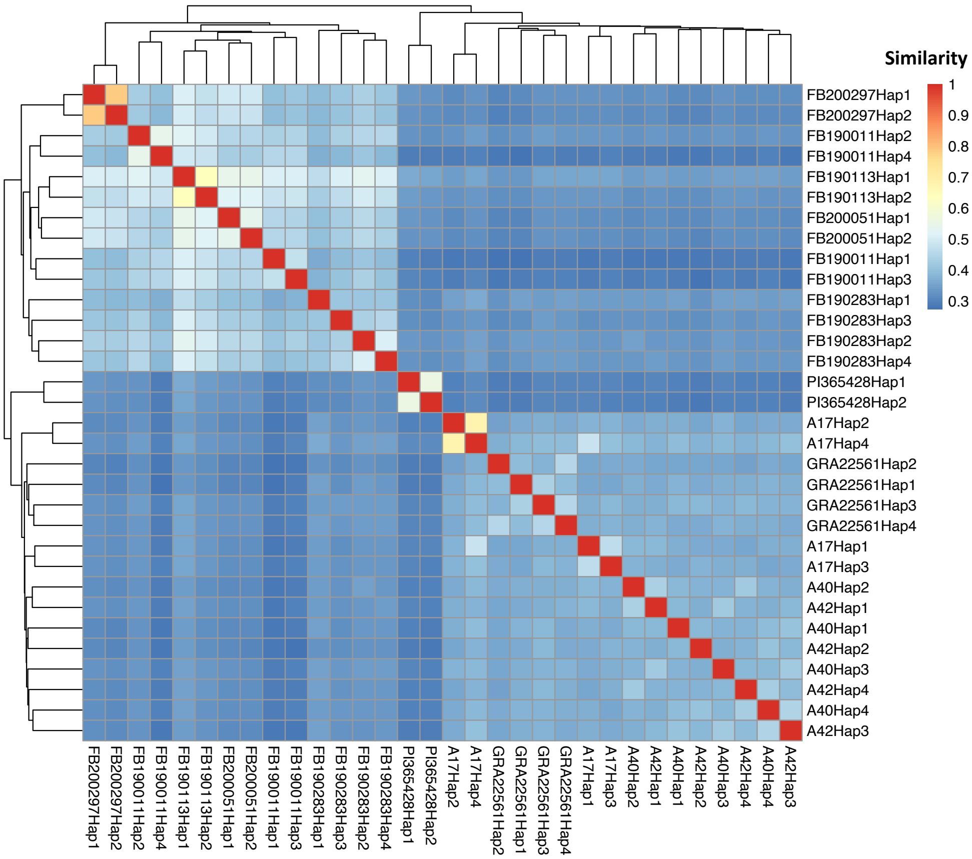

Supplementary Figure 23: Heatmap showing the similarity between genomes in the graph-based pangenome. The similarity is calculated using odgi based on the graph genome. 1 means complete identity, and the smaller the value, the lower the similarity.

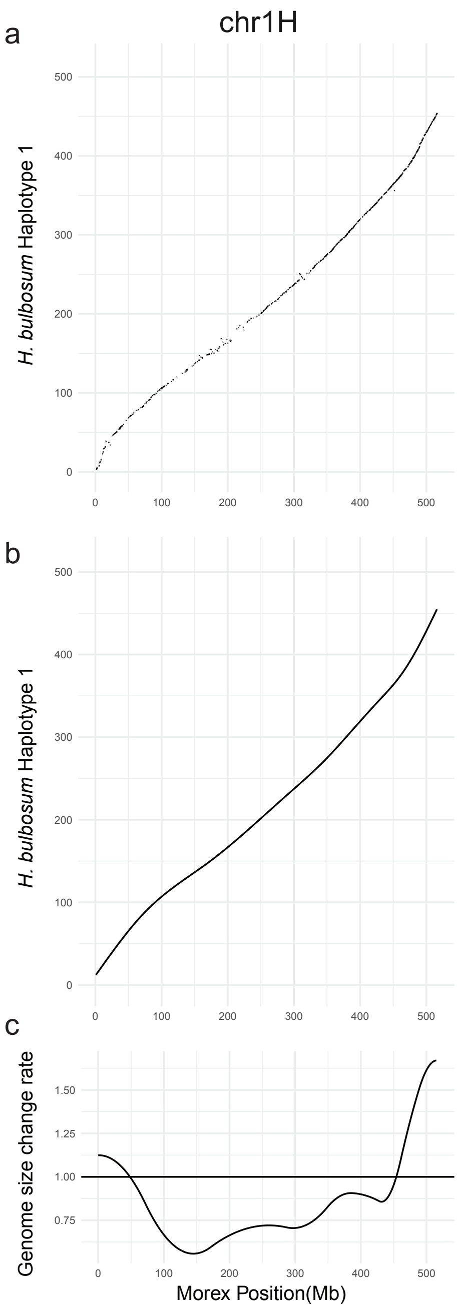

Supplementary Figure 24: Estimation of local genome size change rates based on alignments of chr1H between *H. bulbosum* haplotype 1 and *H. vulgare* cv. Morex. (a) Genome alignment between *H. bulbosum* haplotype 1 and Morex on chromosome 1H. (**b)** Alignments were fitted to a generalized additive model (GAM). **(c)** Estimated genome size change rate along the chromosome. The black horizontal line refers to the genome size change rate equal to 1 (no change).

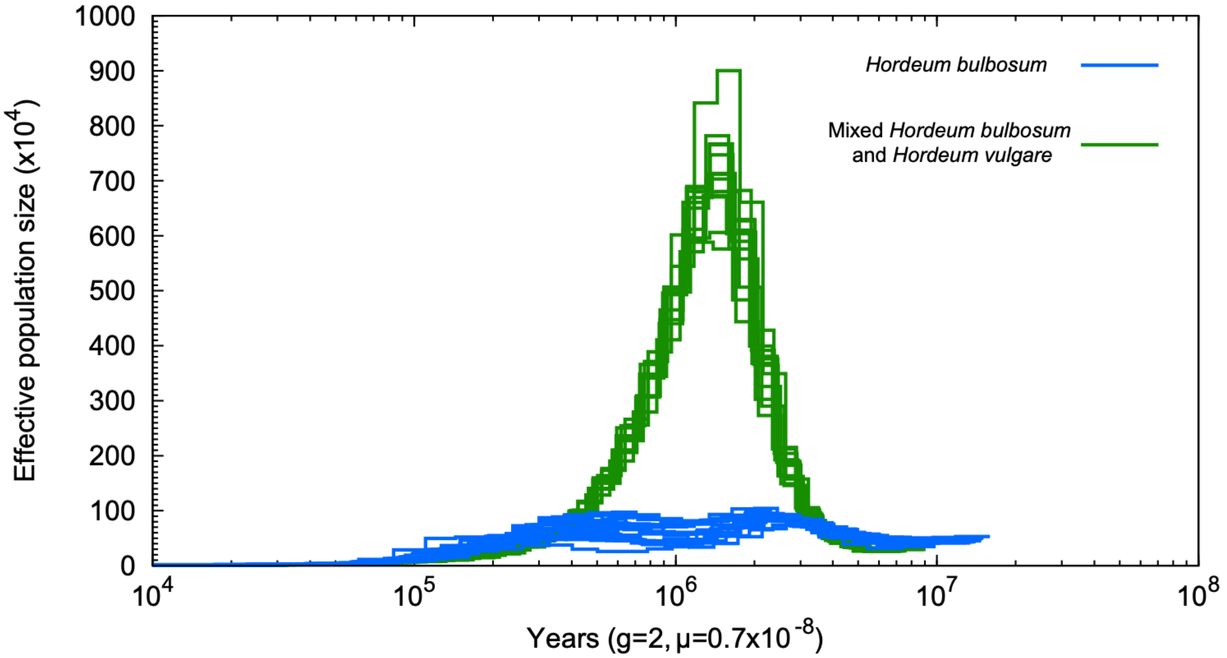

Supplementary Figure 25: Population size trajectories as inferred by PSMC from heterozygous genomes. Blue line refer to *H. bulbosum*; Green line refer to synthetic diploids between *H. bulbosum* and *H. vulgare*.

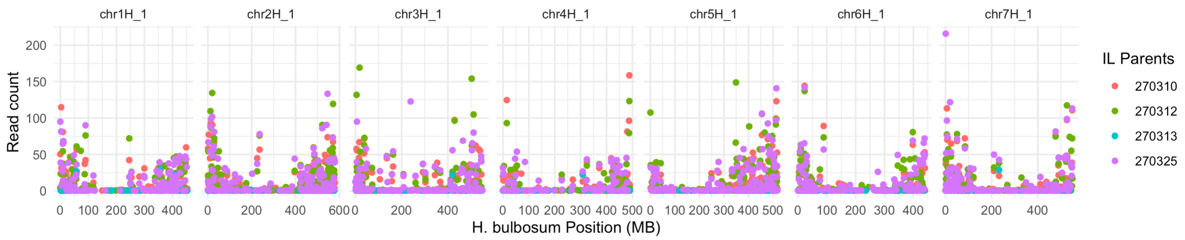

Supplementary Figure 26: Distribution of read counts of barley parents of introgression lines aligned to the *H. bulbosum* genome.

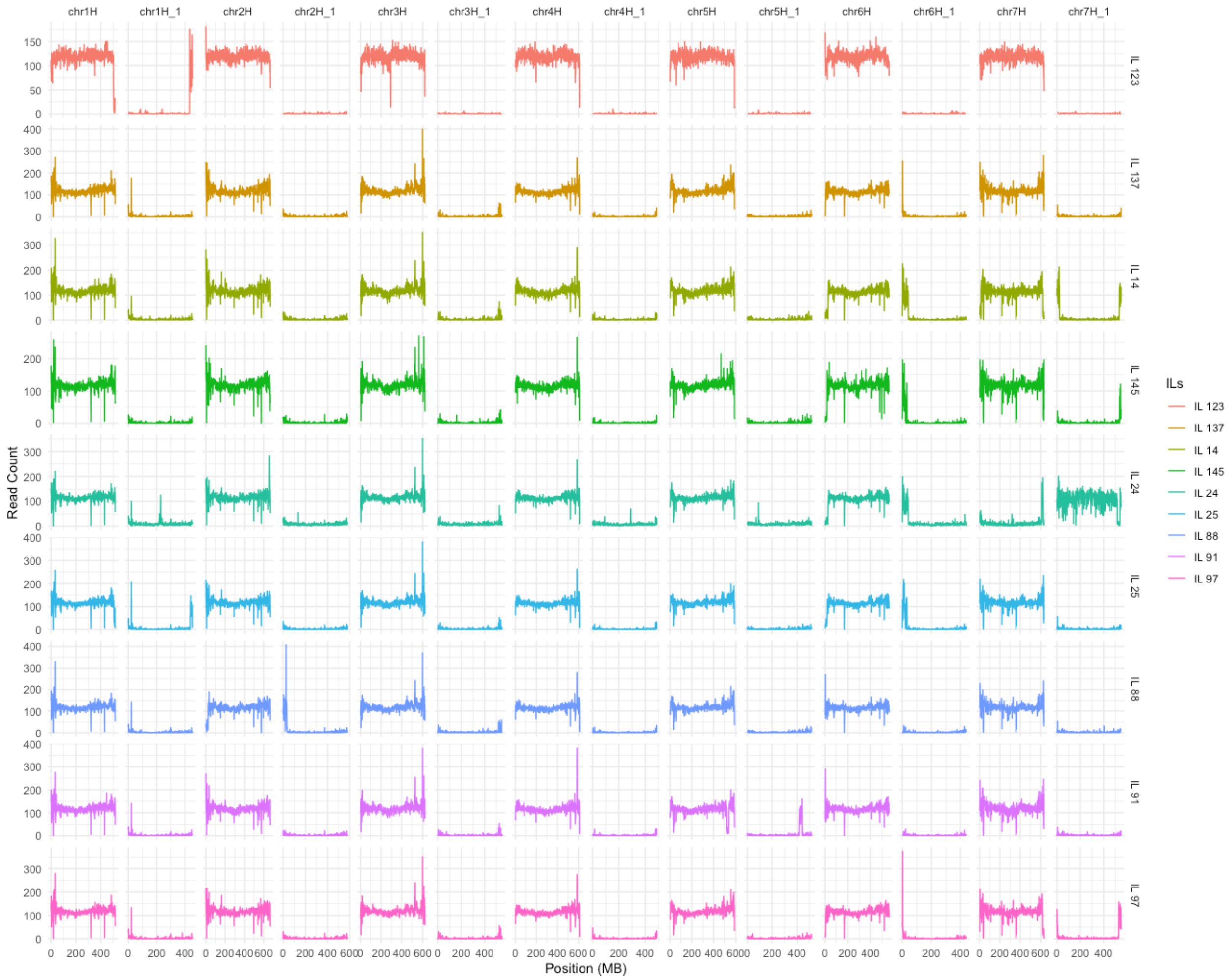

Supplementary Figure 27: Distribution of read counts of HiFi sequences of introgression lines aligned to the *H. bulbosum* and *H. vulgare* concatenated genome. The suffix H_1 marks the *H. bulbosum* genome, H marks the *H. vulgare* genome.

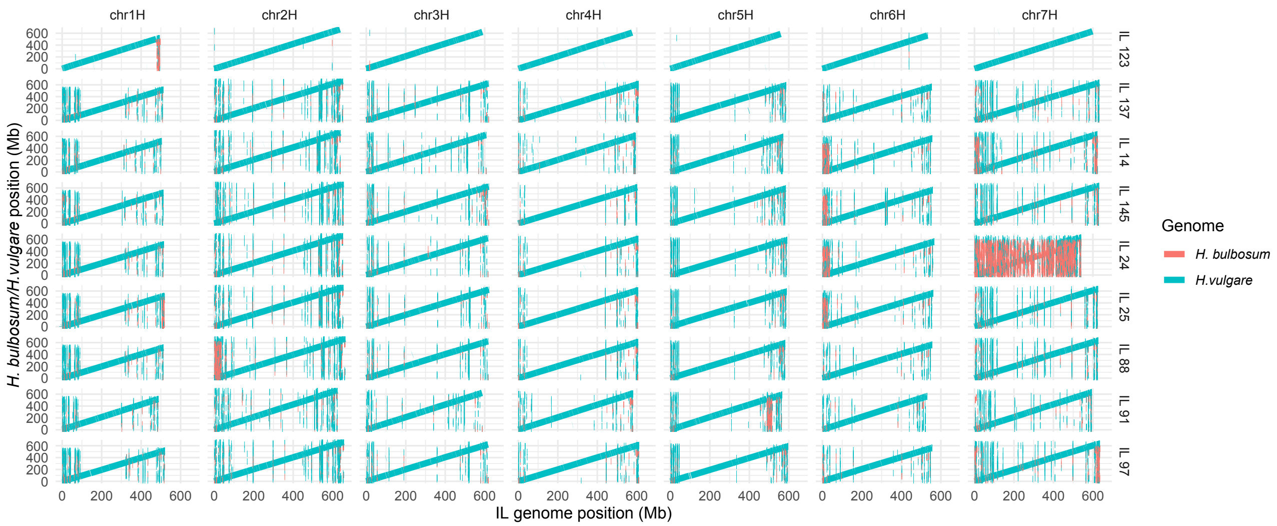

Supplementary Figure 28: Whole-genome alignments of IL genomes against *H. bulbosum* (FB19-011-3 Haplotype 1)/*H. vulgare* (MorexV3) reference. Red is the alignment of FB19-011-3 Haplotype 1 genome and blue is the alignment of MorexV3 genome.

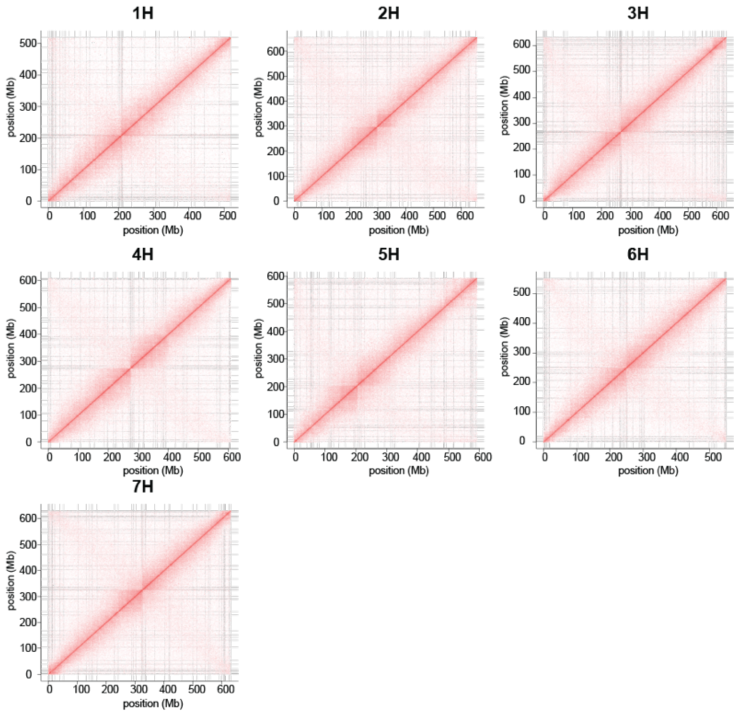

Accession	N50	Assembly size (bp)	unanchored contsize (bp)	Busco (embryophyta_odb9)
JKI-5215	28,413,266	4,271,127,017	76,377,195	96.60%

Supplementary Figure 29: JKI-5215 Hi-C contact matrix and assembly summary statistics.

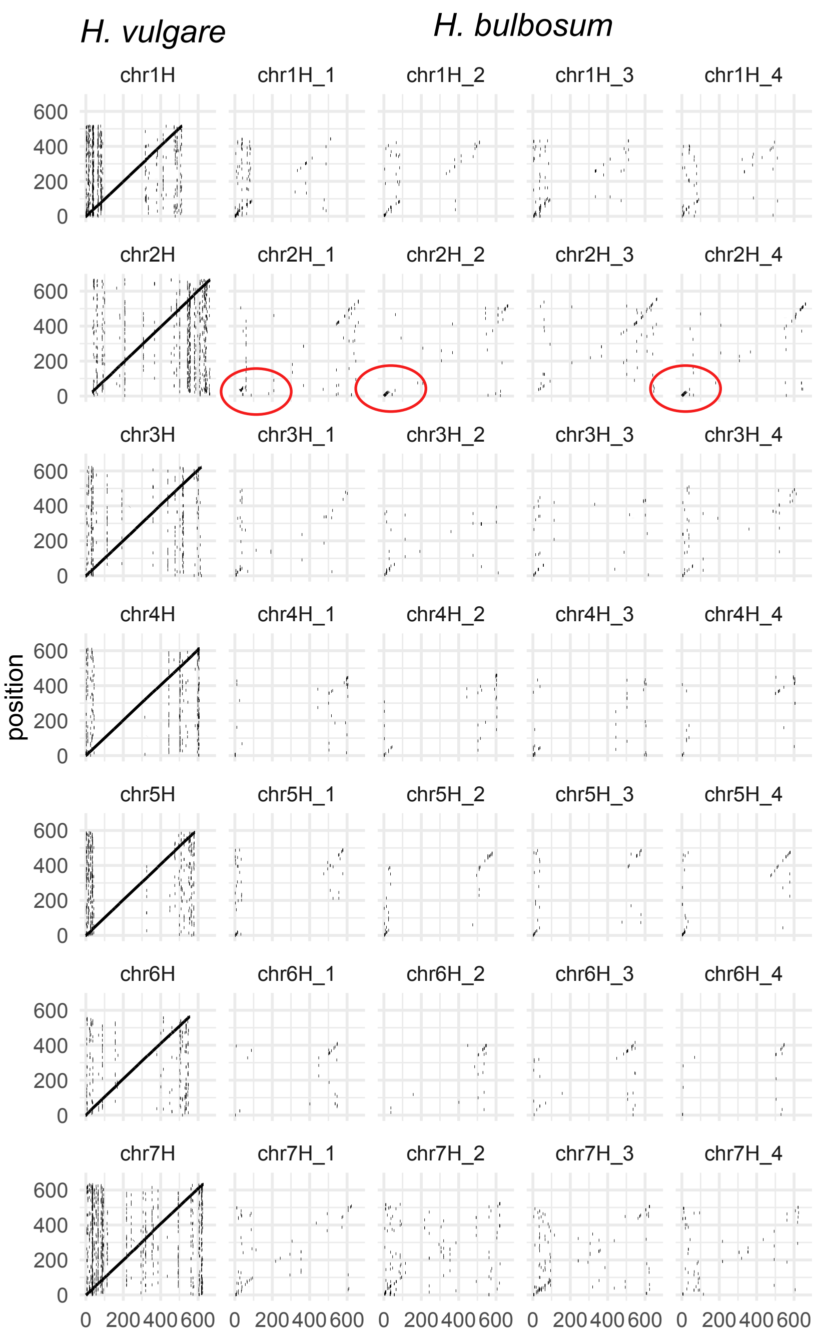

Supplementary Figure 30: Alignment of introgression line IL 88 against a concatenated A17/Morex reference. Red circles mark introgressed segments. Chr2H haplotype 2 and 4 are identical-by-descent in the introgressed region. The entire introgressed segment is the product of a recombination between Chr2H haplotypes 1 and 2/4.

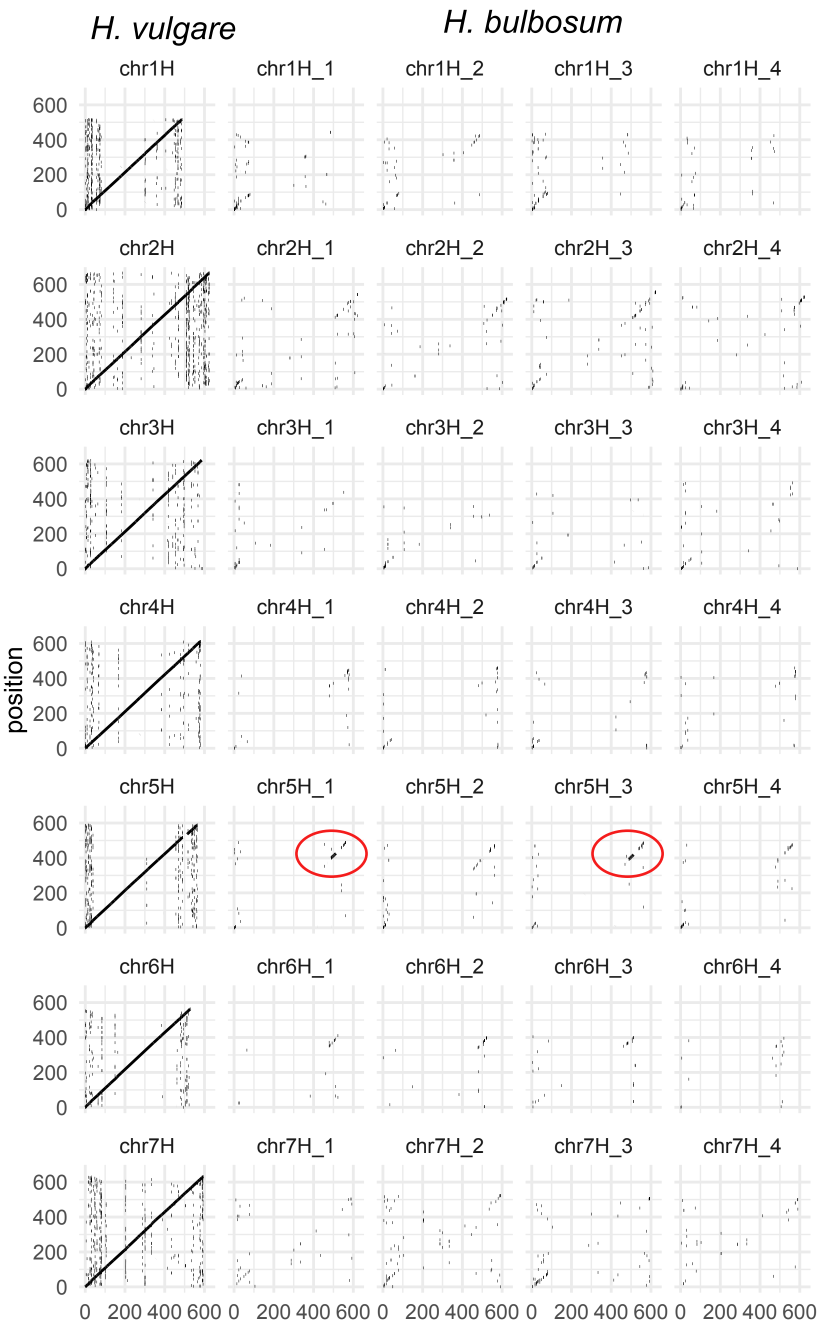

Supplementary Figure 31: Alignment of introgression line IL 145 against a concatenated A17/Morex reference. Red circles mark introgressed segments.

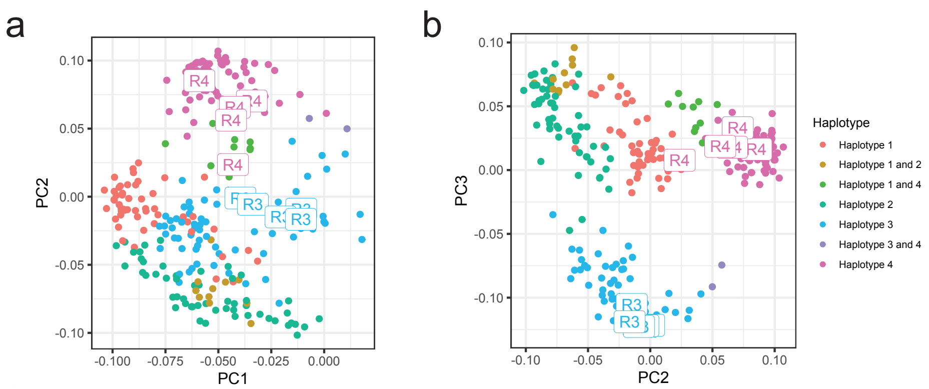

Supplementary Figure 32: A17 assembly separation in the introgressed region of JK-5215. (a) and **(b)** PCA clustering of the matrix of Hi-C links connecting the ends of contigs, i.e. those mapped to within 2 Mb of either contig end. Chromosome 3H of the tetraploid clone A17 is shown. Colors correspond to different haplotypes. R3 corresponds to contigs which are in the region of A17 haplotype 3 collinear to the JKI-5215 introgressed region. R4 corresponds to contigs which are in the region of A17 haplotype4 collinear to the JKI-5215 introgressed region.

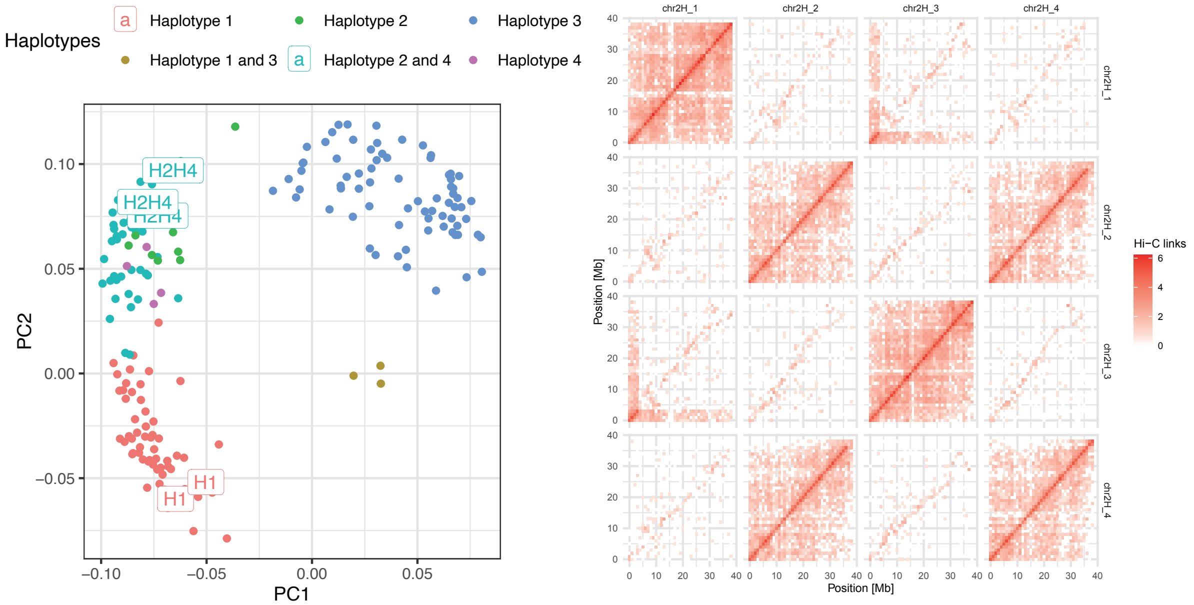

Supplementary Figure 33: A17 assembly separation in the introgressed region of IL 88. (a) PCA clustering of the matrix of Hi-C links connecting the ends of contigs, i.e. those mapped to within 2 Mb of either contig end. Chromosome 3H of the tetraploid clone A17 is shown. Colors correspond to different haplotypes. H2H4 corresponds to contigs which are in the region of A17 haplotype 2 and 4 collinear to the IL 88 introgressed region. H1 corresponds to contigs which are in the region of A17 haplotype 1 collinear to the IL 88 introgressed region. **(b)** Hi-C contact matrix of the region of A17 collinear to the IL 88 introgressed region.

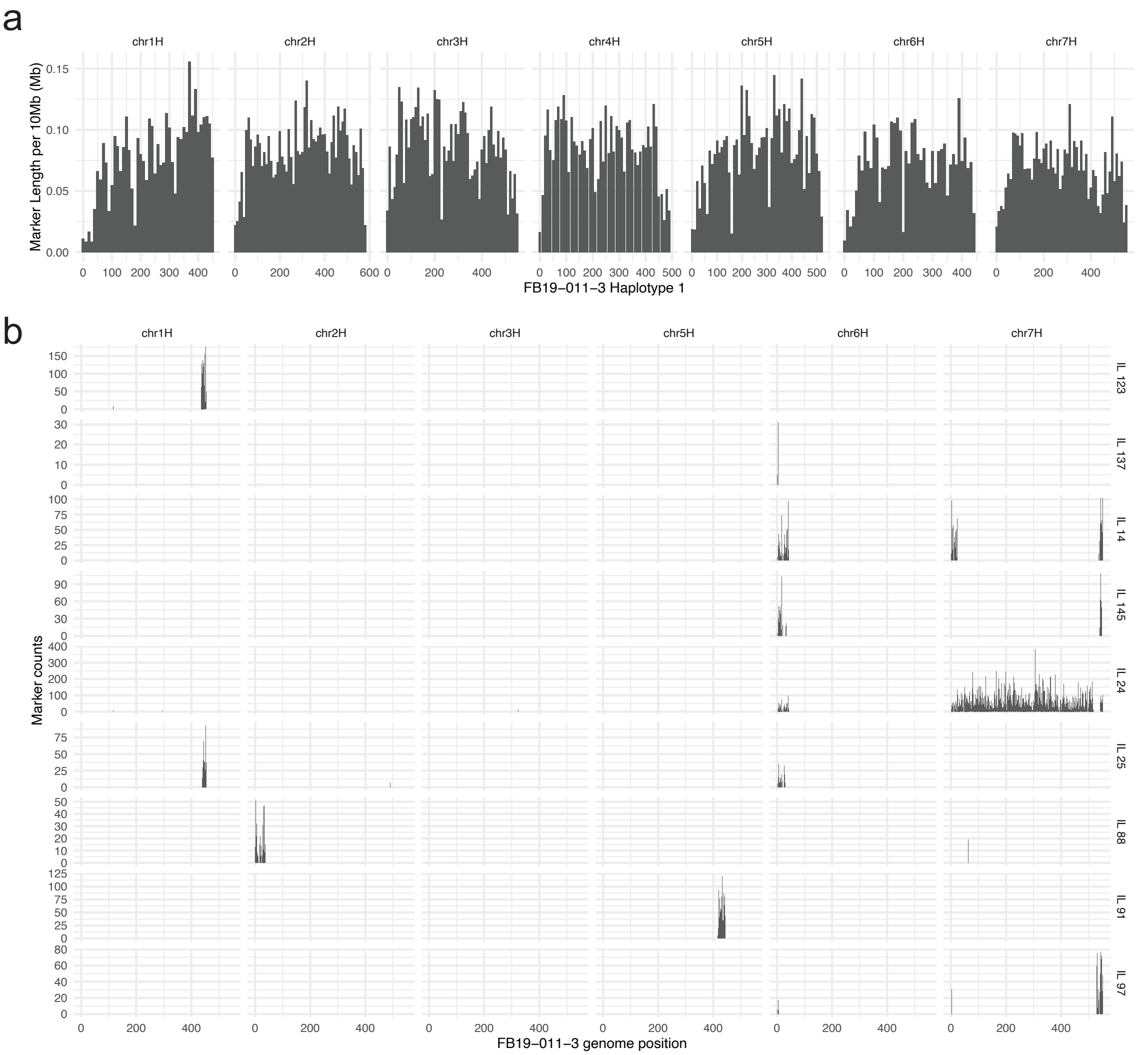

Supplementary Figure 34: Identification of marker sequences to distinguish between *H. bulbosum* and *H. vulgare* chromatin. (a) Distribution of markers on the genome. The sequences are present in the single-copy core genome of 10 *H. bulbosum* individual (32 haplotypes), but do not align to any of the 76 barley assemblies of Jayakodi et al. (2024). **(b)** The mapping of markers to 9 introgression line (IL) genomes.

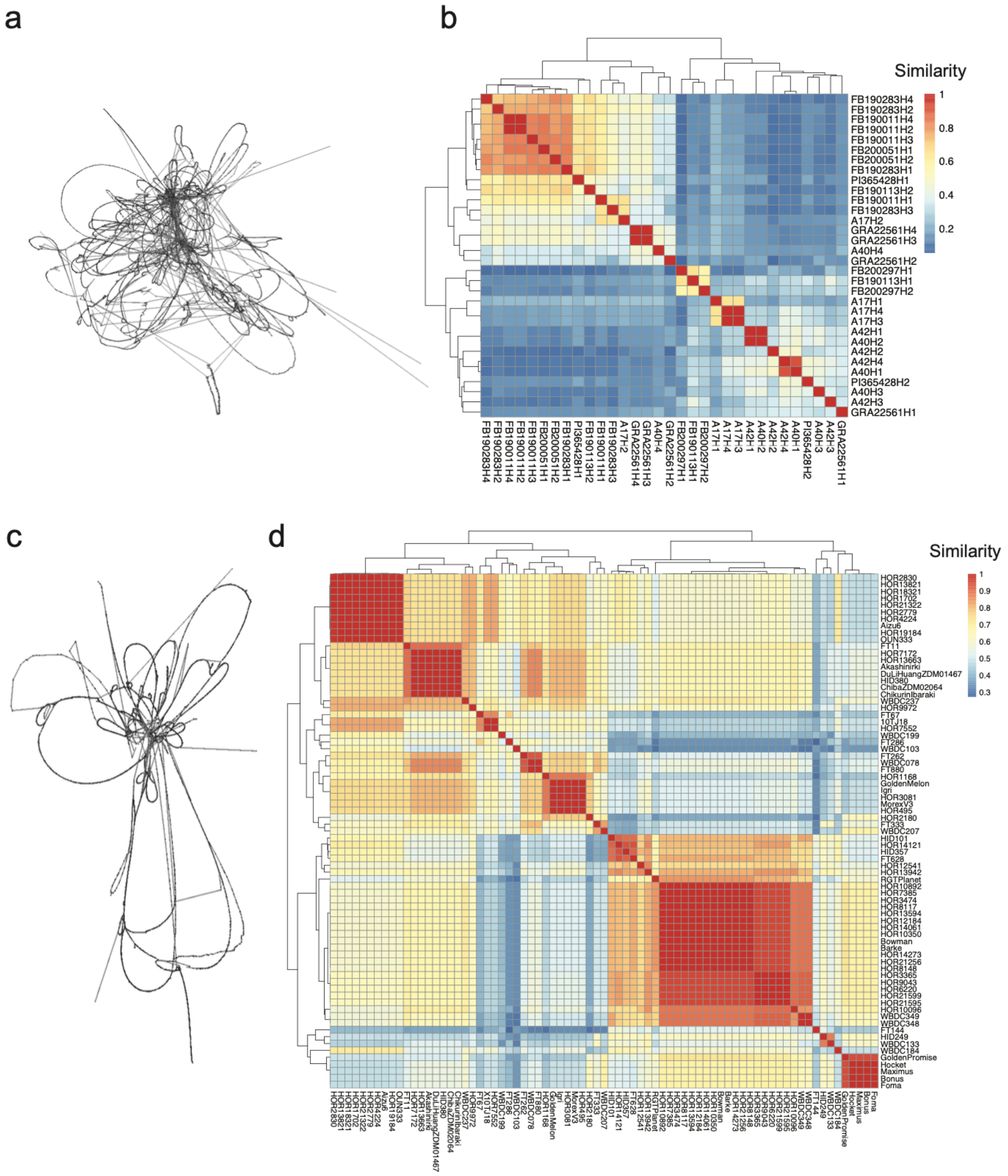

Supplementary Figure 35: Local graph-based pangenome constructed by PGGB in the 100 kb region before and after the *mlo* gene. (a) 2D graph visualization of the *H. bulbosum* graph-based pangenome in *mlo*. **(b)** The heatmap *mlo* in *H. bulbosum* graph-based pangenome. **(c)** 2D graph visualization of barley graph-based pangenome in *mlo*. **(d)** The heatmap of *mlo* in barley graph-based pangenome. The similarity is calculated using odgi based on the graph genome. 1 means complete identity, and the smaller the value, the lower the similarity.

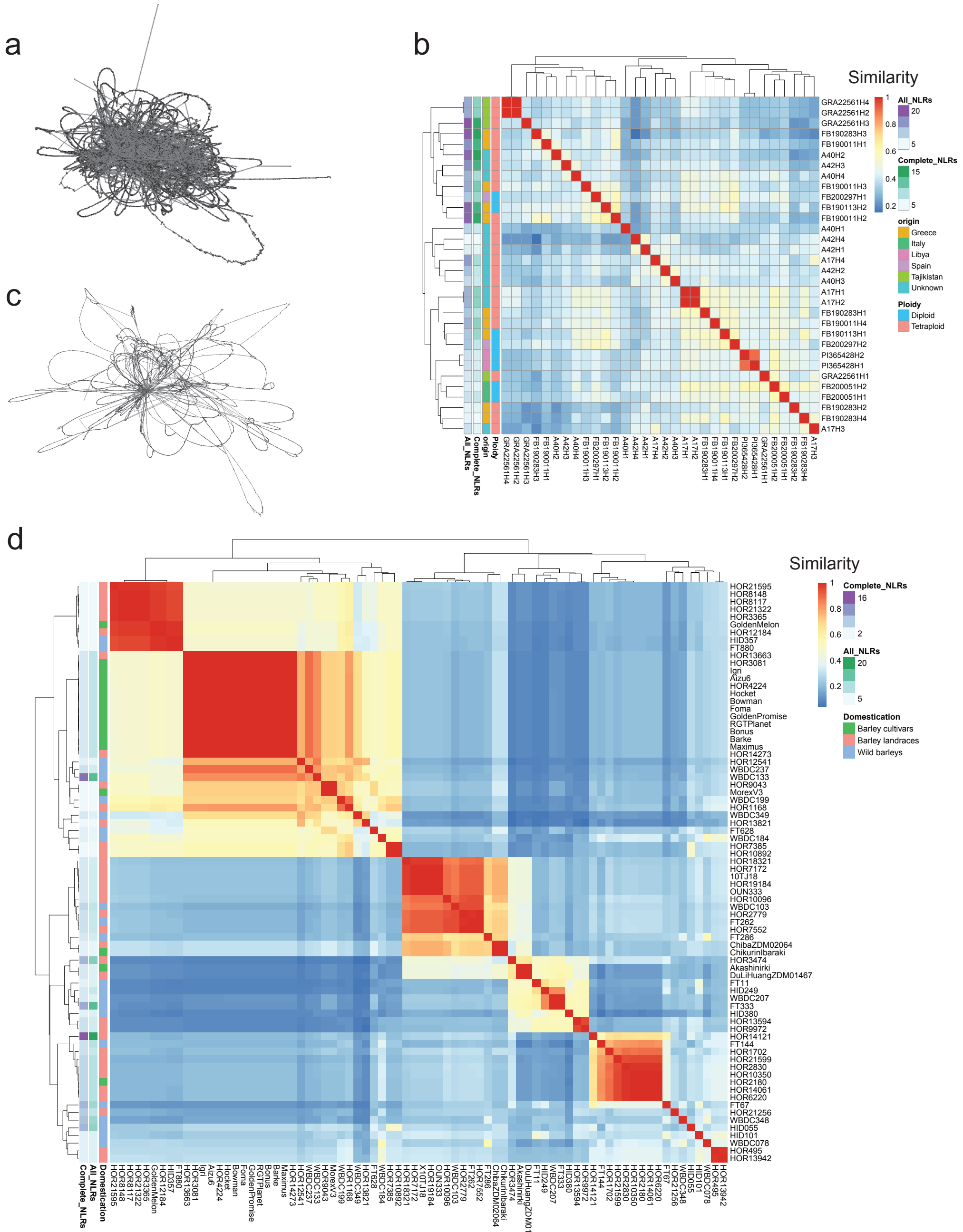

Supplementary Figure 36: Local graph-based pangenome constructed by PGGB in *Ryd4*. (a) 2D graph visualization of *H. bulbosum* graph-based pangenome in *Ryd4*. **(b)** The heatmap of *Ryd4* in *H. bulbosum* graph-based pangenome. **(c)** 2D graph visualization of barley graph-based pangenome in *Ryd4*. **(d)** The heatmap of *Ryd4* in barley graph-based pangenome. The similarity is calculated using odgi based on the graph genome. 1 means complete identity, and the smaller the value, the lower the similarity.

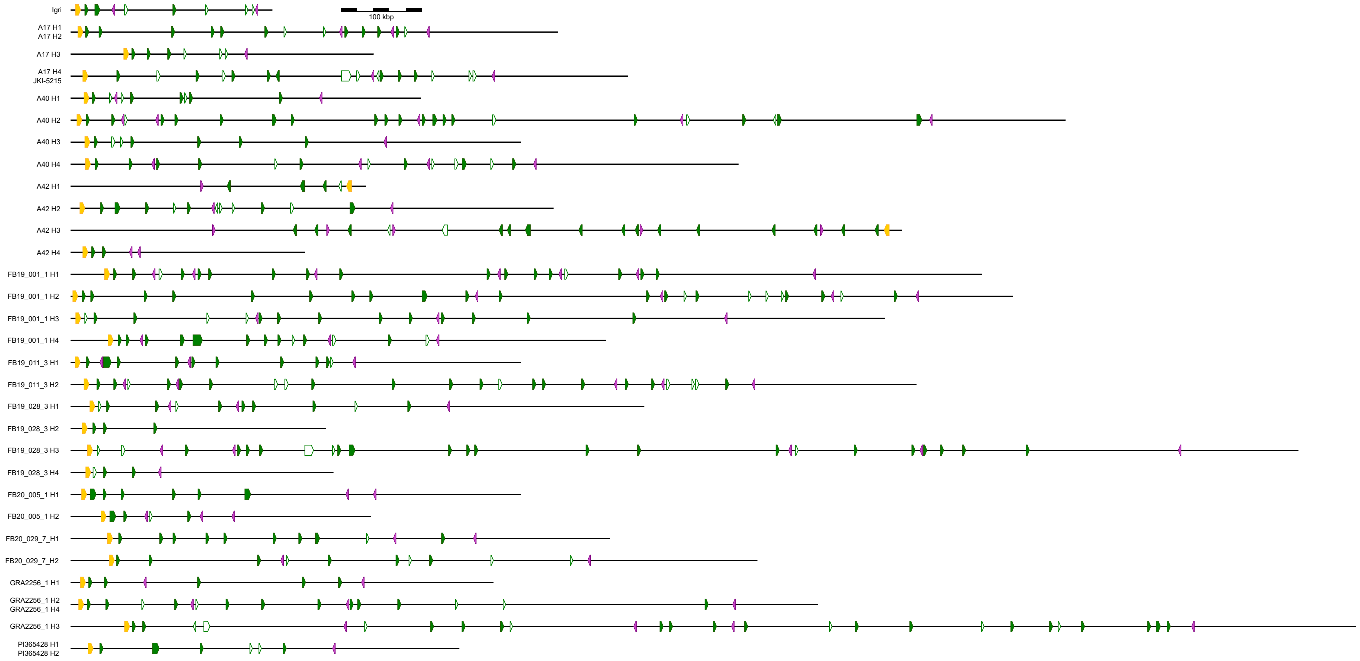

Supplementary Figure 37: Gene content of the *Ryd4^{Hb}* locus in the *H. bulbosum* pangenome. The color code is: yellow – SFGH1, purple – APGG2, green – CC-NBARC-LRR genes as defined by NLR-annotator. Details on the genes are given in **Supplementary Table 16**.

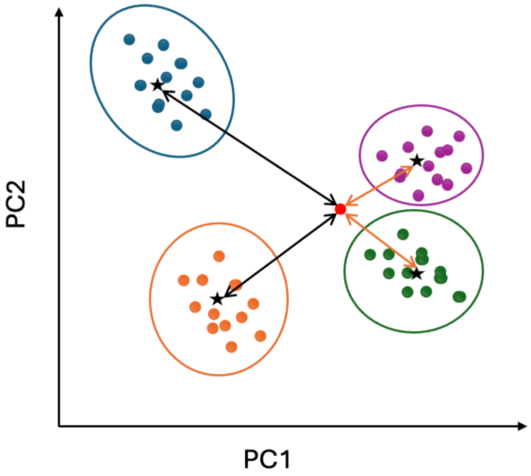

Supplementary Figure 38: Schematic diagram of multi-count contig placement. The points represent each contig involved in phasing. The red point represents multi-count contig. Except for the red point, the remaining points represent single coverage contigs, and different colors represent different haplotypes. The black stars represent the PCA centers of different haplotypes. The line represents the distance from the PCA center to the red points.
