# Supplementary material for: A haplotype-resolved pangenome of the barley wild relative Hordeum bulbosum
Source: Nature. 2025 Jul 9;645(8080):429–38. doi: 10.1038/s41586-025-09270-x (PMC12422954; doi:10.1038/s41586-025-09270-x)
Supplement: Supplementary file 2 — Reporting Summary [file 41586_2025_9270_MOESM2_ESM.pdf]

Corresponding author(s): M. Mascher, N. Stein, F. Blattner

Last updated by author(s): 27.05.2025

Reporting Summary

Nature Portfolio wishes to improve the reproducibility of the work that we publish. This form provides structure for consistency and transparency in reporting. For further information on Nature Portfolio policies, see our Editorial Policies and the Editorial Policy Checklist.

Please do not complete any field with "not applicable" or n/a. Refer to the help text for what text to use if an item is not relevant to your study.
For final submission: please carefully check your responses for accuracy; you will not be able to make changes later.

Statistics

For all statistical analyses, confirm that the following items are present in the figure legend, table legend, main text, or Methods section.

n/a	Confirmed	
☐	☒	The exact sample size (*n*) for each experimental group/condition, given as a discrete number and unit of measurement
☐	☒	A statement on whether measurements were taken from distinct samples or whether the same sample was measured repeatedly
☐	☒	The statistical test(s) used AND whether they are one- or two-sided<br>*Only common tests should be described solely by name; describe more complex techniques in the Methods section.*
☒	☐	A description of all covariates tested
☐	☒	A description of any assumptions or corrections, such as tests of normality and adjustment for multiple comparisons
☐	☒	A full description of the statistical parameters including central tendency (e.g. means) or other basic estimates (e.g. regression coefficient) AND variation (e.g. standard deviation) or associated estimates of uncertainty (e.g. confidence intervals)
☒	☐	For null hypothesis testing, the test statistic (e.g. *F*, *t*, *r*) with confidence intervals, effect sizes, degrees of freedom and *P* value noted<br>*Give P values as exact values whenever suitable.*
☒	☐	For Bayesian analysis, information on the choice of priors and Markov chain Monte Carlo settings
☒	☐	For hierarchical and complex designs, identification of the appropriate level for tests and full reporting of outcomes
☒	☐	Estimates of effect sizes (e.g. Cohen's *d*, Pearson's *r*), indicating how they were calculated

Our web collection on statistics for biologists contains articles on many of the points above.

Software and code

Policy information about availability of computer code

Data collection	Data collection used proprietary software of PacBio and Illumina for raw data processing.
Data analysis	Multiple published software packages were used in the analysis including: ADMIXTURE (v1.3.0), ASMAP(v1.0-2), Augustus (v3.3.3), Badread (v0.4.1), BBMap (v37.93), BCFtools (v1.9), BEDTools (v2.30.0), BLAST (v2.13.0), bwa (v0.7.17-r1188), Canu (v2.1.1), CD-HIT, cutadapt (v1.15), DeepVariant (v1.6.0), EVidenceModeller (v2.1.0), Extensive de-novo TE Annotator (EDTA) (v1.9.0), FASTME (v2.1.5), FastTree (v2.1.11), GENESPACE (v1.2.3), GeSeq (https://chlorobox.mpimp-golm.mpg.de/geseq.html), gfatools (v0.4-r179), glnexus_cli (v1.4.1), GMAP (v2018-07-04), hifiasm (v0.13-r308), IntroBlocker (v1.0.0), IQ-TREE (v2.2.0-beta), iTOL (https://itol.embl.de/) , LASTZ (v1.04.03), MAFFT (v7.490), Merqury (v1.3), mgcv (v1.8-42), Mikado (v2.3.4), Minigraph-Cactus pangenome pipeline (v2.6.5), minimap2 (v2.24-r1122 or 2.26-r1175), miniprot (0.11-r234), MMSeq2 (v2), Mummer4 (v4.0.0beta2), MUSCLE, NLR annotator (v2), Novosort (v3.09.01), ODGI (v0.8.6-2), PASA (v2.5.3), pbmm2 (v1.13.1), PGGB (v0.5.3), PLINK (v1.90b6.9), PSMC (V0.6.5-r67), RagTag (v2.1.0), SAMtools (v1.16.1), seqkit (v0.9.1), SIFT, SNPrelate(v1.16.0) splitfa, STAR (v2.7.8a), StringTie (v2.1.5), svim (v1.4.2), SyRI (v1.6), TRITEX pipeline (https://tritexassembly.bitbucket.io/), vg (v1.49.0), wfmash (v0.8.2), wgsim (v0.3.1-r13). Genome assembly and data analysis (https://github.com/jia-wu-feng/Pan_Bulbosum), Gene projection (https://github.com/GeorgHaberer/gene_projection/tree/main/panhordeum), Analyis of orthologous groups (https://github.com/PGSB-HMGU/hbulbosum/tree/main)

For manuscripts utilizing custom algorithms or software that are central to the research but not yet described in published literature, software must be made available to editors and reviewers. We strongly encourage code deposition in a community repository (e.g. GitHub). See the Nature Portfolio guidelines for submitting code & software for further information.

Data

Policy information about availability of data

All manuscripts must include a data availability statement. This statement should provide the following information, where applicable:

- Accession codes, unique identifiers, or web links for publicly available datasets
- A description of any restrictions on data availability
- For clinical datasets or third party data, please ensure that the statement adheres to our policy

All the sequence data collected in this study have been deposited at the European Nucleotide Archive (ENA) under BioProjects PRJEB65276 (Genome assemblies, transcriptome Illumina data, GBS data) and PRJEB65918 (pollen nuclei single-cell sequencing data). Accession codes for individual genotypes are listed in supplementary tables: Supplementary Table 1, 2 and 7 (pangenome assemblies and associated raw data), Supplementary Table 6 (pollen single-nuclei sequencing data), Supplementary Table 12 (GBS). The assemblies and annotations are available for download from https://galaxy-web.ipk-gatersleben.de/libraries/folders/Fb701da857886499b. The variant matrix and pangenome graphs were deposited under a DOI (http://dx.doi.org/10.5447/ipk/2025/4) at the Plant Genomics and Phenomics Research Data Repository. TREP database (https://trep-db.uzh.ch/)

Research involving human participants, their data, or biological material

Policy information about studies with human participants or human data. See also policy information about sex, gender (identity/presentation), and sexual orientation and race, ethnicity and racism.

Reporting on sex and gender	not applicable
Reporting on race, ethnicity, or other socially relevant groupings	not applicable
Population characteristics	not applicable
Recruitment	not applicable
Ethics oversight	*Identify the organization(s) that approved the study protocol.*

Note that full information on the approval of the study protocol must also be provided in the manuscript.

Field-specific reporting

Please select the one below that is the best fit for your research. If you are not sure, read the appropriate sections before making your selection.

☒ Life sciences ☐ Behavioural & social sciences ☐ Ecological, evolutionary & environmental sciences

For a reference copy of the document with all sections, see nature.com/documents/nr-reporting-summary-flat.pdf

Life sciences study design

All studies must disclose on these points even when the disclosure is negative.

Sample size	Population structure of a diversity panel was analyzed. Representative accessions from different populations, geographic origins and ploidy levels were chosen.
Data exclusions	No data were excluded.
Replication	DNA/RNA was extracted from verified clones of the same genotypes.
Randomization	Genome assembly and analysis were conducted on a individual H. bulbosum plant, thus randomization is not necessary.
Blinding	Genome assembly and analysis were conducted on a individual H. bulbosum plant, thus randomization is not necessary.

Reporting for specific materials, systems and methods

We require information from authors about some types of materials, experimental systems and methods used in many studies. Here, indicate whether each material, system or method listed is relevant to your study. If you are not sure if a list item applies to your research, read the appropriate section before selecting a response.

Materials & experimental systems

n/a	Involved in the study
☒	☐ Antibodies
☒	☐ Eukaryotic cell lines
☒	☐ Palaeontology and archaeology
☒	☐ Animals and other organisms
☒	☐ Clinical data
☒	☐ Dual use research of concern
☐	☒ Plants

Methods

n/a	Involved in the study
☒	☐ ChIP-seq
☒	☐ Flow cytometry
☒	☐ MRI-based neuroimaging

Dual use research of concern

Policy information about dual use research of concern

Hazards

Could the accidental, deliberate or reckless misuse of agents or technologies generated in the work, or the application of information presented in the manuscript, pose a threat to:

No	Yes
☒	☐ Public health
☒	☐ National security
☒	☐ Crops and/or livestock
☒	☐ Ecosystems
☒	☐ Any other significant area

Experiments of concern

Does the work involve any of these experiments of concern:

No	Yes
☒	☐ Demonstrate how to render a vaccine ineffective
☒	☐ Confer resistance to therapeutically useful antibiotics or antiviral agents
☒	☐ Enhance the virulence of a pathogen or render a nonpathogen virulent
☒	☐ Increase transmissibility of a pathogen
☒	☐ Alter the host range of a pathogen
☒	☐ Enable evasion of diagnostic/detection modalities
☒	☐ Enable the weaponization of a biological agent or toxin
☒	☐ Any other potentially harmful combination of experiments and agents

Plants

Seed stocks	The genebanks of IPK Gatersleben and USDA-ARS for provided seeds for this study. Collecting and exchange of seeds followed the regulations of the respective countries and Nagoya agreements for material exchange. One herbarium voucher for each population was deposited in the herbarium of the IPK Gatersleben (GAT).
Novel plant genotypes	No novel seed stock were generated.
Authentication	NA

This checklist template is licensed under a Creative Commons Attribution 4.0 International License, which permits use, sharing, adaptation, distribution and reproduction in any medium or format, as long as you give appropriate credit to the original author(s) and the source, provide a link to the Creative Commons license, and indicate if changes were made. The images or other third party material in this article are included in the article's Creative Commons license, unless indicated otherwise in a credit line to the material. If material is not included in the article's Creative Commons license and your intended use is not permitted by statutory regulation or exceeds the permitted use, you will need to obtain permission directly from the copyright holder. To view a copy of this license, visit http://creativecommons.org/licenses/by/4.0/
