# Supplementary material for: A haplotype-resolved pangenome of the barley wild relative Hordeum bulbosum
Source: Nature. 2025 Jul 9;645(8080):429–38. doi: 10.1038/s41586-025-09270-x (PMC12422954; doi:10.1038/s41586-025-09270-x)
Supplement: Supplementary file 4 — Supplementary Tables 1–18. [file 41586_2025_9270_MOESM4_ESM.zip › 2024-01-01779E-s4/SupplementaryTableLegends.docx]

**Supplementary Tables (combined in one XLSX file)**

**Supplementary Table 1:** Summary statistics and accession codes of HiFi and Hi-C data.

**Supplementary Table 2:** Summary statistics and accession codes of 10 genomes.

**Supplementary Table 3:** Optical map statistics.

**Supplementary Table 4:** Sequence assembly and hybrid scaffold statistics for *H. bulbosum* FB19-011-3 haplotype 1.

**Supplementary Table 5:** Sequence assembly and hybrid scaffold statistics for *H. bulbosum* FB19-011-3 haplotype 2.

**Supplementary Table 6:** Summary statistics and accession codes of pollen sequencing.

**Supplementary Table 7:** Summary statistics and accession codes of RNA-Seq and Iso-Seq data.

**Supplementary Table 8:** Summary statistics of 10 genome assemblies.

**Supplementary Table 9:** Haplotype-level summary statistics for *H. bulbosum* genomes.

**Supplementary Table 10:** Summary of gene annotation.

**Supplementary Table 11:** Summary of the transposable element annotation.

**Supplementary Table 12:** Summary statistics and accession codes of GBS data.

**Supplementary Table 13:** Map of donor segments in *H. vulgare*-*H. bulbosum* ILs.

**Supplementary Table 14:** Summary statistics of 9 introgression line genome assemblies.

**Supplementary Table 15:** Summary of local graph-based pangenome constructed by PGGB.

**Supplementary Table 16:** List of putative candidate gene in the *Ryd4^Hb^* interval.

**Supplementary Table 17:** List of putative candidate genes for the sublethality factor.

**Supplementary Table 18:** Summary of simulate long reads alignment.
